# Research on differential game of platform corporate social responsibility governance strategy considering user and public scrutiny

**DOI:** 10.1371/journal.pone.0301632

**Published:** 2024-04-26

**Authors:** Yongquan Guo, Hua Zou, Zhu Liu, Baotong Liu

**Affiliations:** 1 School of Management, Shenyang University of Technology, Shenyang, China; 2 Shenyang Normal University, Shenyang, China; King Abdulaziz University, SAUDI ARABIA

## Abstract

The development of digital technology and the sharing economy has extended corporations’ innovative activities beyond the corporation’s boundaries, so it has become more urgent to govern the lack of social responsibility and alienation of platform corporations from the perspective of social agents. First, the platform’s CSR classification and social responsibility governance’s main content are analyzed in this research. Then, this study uses government agencies, platform corporations, users, and the public as governance subjects and compares governance decisions with and without public and user oversight. Finally, the optimal balance strategy for each governing subject, the optimal trajectory of governance volume, and the trajectory of total revenue are obtained. The study found that: 1) Public and user supervision can improve the governance volume while encourage the governance motivation of government agencies and platform corporations. 2) The level of user supervision effort has a greater impact on the total governance revenue than public supervision. 3) The revenue of the system and the governance volume are greater in a centralized decision-making process, indicating that those involved should co-operate in governance based on the principle of mutual benefit. 4) The platform corporation has an incompatible but unified relationship between its social duty and financial success.

## Introduction

With the acceleration of the development of new-generation information technology and market demand, the digital transformation of traditional enterprises has become a new choice to reshape the competitiveness of corporations in order to meet the fast-changing market forms. On the one hand, traditional enterprises are transforming their organizational structure to become platform enterprises, and on the other hand, they are using digital functional platforms to connect with a wide range of participants to form innovation ecosystems. It is because of the platform strategy of traditional manufacturing corporations that some have gained outstanding economic benefits. For example, Hai’er Group has leveraged its platform strategy to create organizational reforms. In 2018, Hai’er Group achieved a global turnover of 266.1 billion RMB, successfully transitioning from a manufacturing company to a platform company. Scholars have studied the impact of CSR on cross-border mergers and acquisitions (M&A) by Chinese firms and concluded that firms with high CSR are more likely to succeed in cross-border M&A [1]. It can be seen that corporate social responsibility has a positive impact on improving corporate performance. However, while corporations concentrate on business practices and economic functions, they must also pay attention to social concerns and actively assume social responsibility [2]. Corporate social responsibility (CSR) is the role of a corporation in serving the public interest through relevant policies and practices [3]. Platform CSR emphasizes that corporations must be accountable for the interests of others, including the natural environment, the social environment, and other partners. However, there is a significant mismatch between platform corporations’ economic and social responsibilities. On the one hand, with platform corporations’ rapid development and growth, these corporations promote the economic development of society by taking advantage of their scale, network, and innovation. Platform corporations have helped advance technology, creating jobs. Also, platform corporations’ public welfare activities have become a significant motivator for charity. On the other hand, the lack of social responsibility and alienation in recent years are common. Illegal data acquisition by platform businesses, unfair competition, and platform coercion are common industry challenges. If these violations are not addressed, they will seriously impede social and economic development [4]. Each year, China’s state-owned enterprises suffer economic losses of up to 600 billion yuan due to credit deficiencies. The Chinese government also attaches great importance to platform corporate social responsibility governance. It has issued documents such as "the Regulations on the Security Protection of Critical Information Infrastructure." In "Several Opinions on Promoting the Healthy and Sustainable Development of the Platform Economy," it is pointed out that it is necessary to establish a sound platform economy governance system and promote the healthy and sustainable development of the platform economy.

As the importance of CSR has increased, CSR governance has attracted the attention of scholars in several fields. Corporate social responsibility goes beyond the traditional view of maximizing profits and focuses on the company’s responsibility towards its employees [5], leaders [6], and other stakeholders [7]. A number of existing studies have argued that the social responsibility of traditional businesses is primarily concerned with the protection of the natural environment [8], focusing on the combined effects of corporate behavior on business and the environment [9]. However, the application of digital technology and the transformation of conventional corporations have also changed the connotation of corporate social responsibility, and it needs to be redefined. In the research on corporate social responsibility, terms such as citizens, stakeholders, ethics [10], sustainability [11, 12], competition and Complementation [13], low carbon [14] are widely used to describe social responsibility. From these keywords, the stakeholder perspective is essential for studying corporate social responsibility governance. However, these subjects are less often considered as a whole in existing studies, and the governance roles of the public and users are less often analyzed.

Therefore, this article first analyzes the existing research status and the content of platform corporate social responsibility (in Literature review); The Problem description and research framework section examines the stakeholders and relationships between stakeholders in governance and then establishes a differential game model of the system based on the relationships between stakeholders (in Model construction), obtains the optimal solution of the system, and determines the strength of the role of the public and users in governance effectiveness. The Comparative analysis and Numerical simulation section validated the results using simulation. Finally, governance strategies and suggestions were proposed.

## Literature review

### The connotations of platform CSR social responsibility

**Fig 1:**
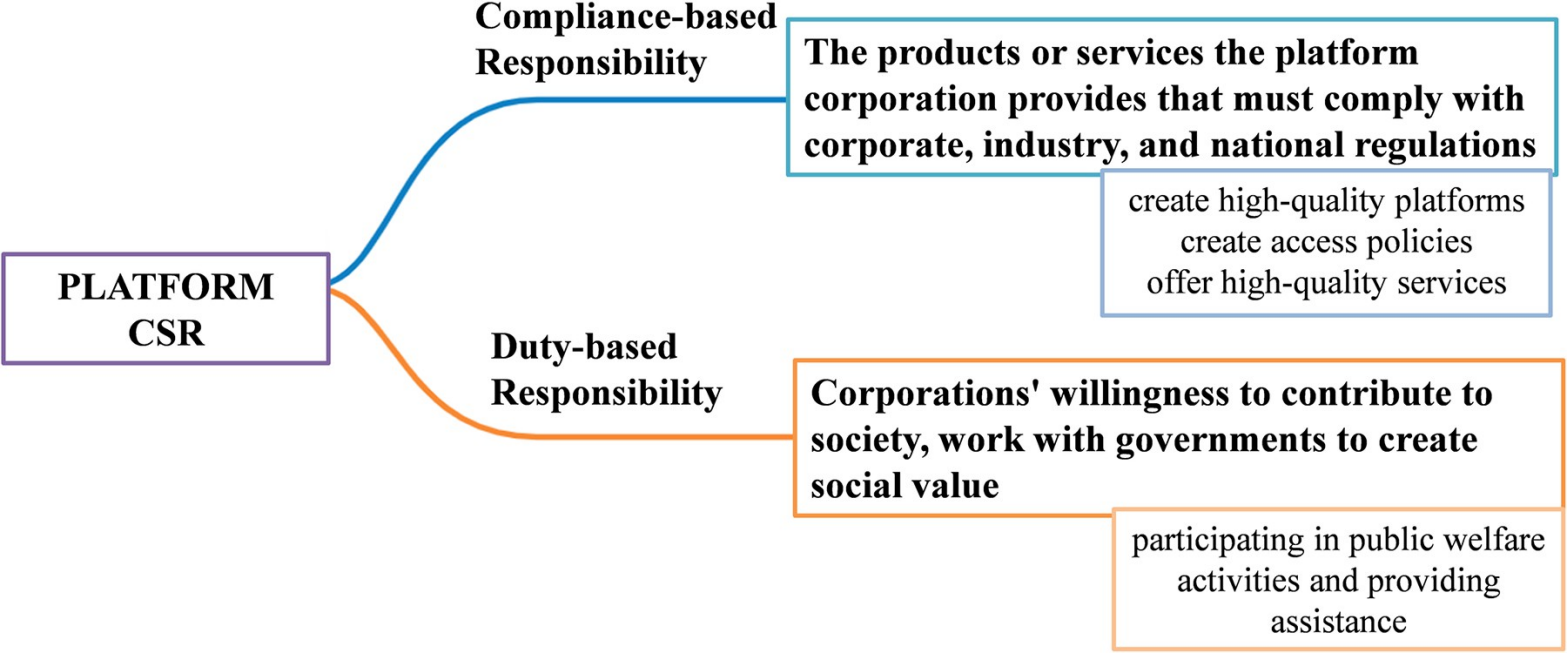
Classification of platform CSR.

In terms of the connotations of social responsibility, social responsibility of corporations includes legal operation, environmental protection, recycling of resources [15] and other aspects. The deep integration of the new generation of information technology and traditional industries makes the platform for enterprise social responsibility show different characteristics from traditional enterprises. The platform’s CSR is increasingly focused on data management. According to Li and Chen, the platform of corporate social responsibility is classified into two categories: compliance-based responsibility and duty-based responsibility [16, 17]. Compliance-based responsibility refers to the products or services the platform corporation provides that must comply with corporate, industry, and national regulations [18]. The corporation’s actions, such as manufacturing process safety, must not violate public order or ethics. The compliance-based responsibility of platform companies therefore means that platform corporations should create high-quality platforms, formulate access policies, and provide quality services for bilateral users. Due to their resource allocation function, platform companies also need to ensure the standardization of cooperation between supply and demand parties. Duty-based responsibility is indicated by the corporations’ willingness to contribute to society. For example, platform corporations collaborate with the government to engage in public welfare activities and provide services to other collaborators to create social value. The categories and content of the Platform’s CSR is shown in Fig 1.

### Governance subjects of platform CSR

In terms of governance subjects, in the current study of corporate social responsibility governance, most researchers believe that stakeholders in governance include shareholders, directors [19, 20], employees, and other internal company members. For example, examining the relationship between CSR and staff innovation performance [21, 22] or the impact of business founders on CSR [23]. Many researchers believe that external partners are also essential factors to consider in the governance of platforms CSR. Stakeholders such as supply chain corporations [24], customers [25], and governments [26] can all play a role in fulfilling corporate social responsibilities. And other researchers believe that the primary requirement for successful collaboration involves a broad spectrum of all parties affected by or concerned about the issue [27], collective responsibility plays a vital role in achieving the sustainable development of an organization [28]. The absence and alienation of platform CSR can jeopardize the interests of numerous subjects. Consequently, platform corporations must prioritize the common interests of their stakeholders to win together [29].

### Research methods of platform CSR governance

In terms of research methods, the existing research mainly uses empirical. For example, the scholar uses empirical research to explore the impact of evaluating ESG on corporate green innovation [30], and scholars have also used the research methods to construct and verify an indicator system for the social responsibility of small and medium-sized enterprises [31]. Some scholars have used qualitative research methods to conduct research on the content of corporate social responsibility. For example, Scholars have used qualitative research methods to construct an indicator system for the social impact of shared platforms [32]. However, such studies are static and cannot describe the dynamics of the governance process over time. Therefore, there is a need for a research method that can describe the change of the process and the influence of the subject, and the differential game method can portray the change process of the problem, so this method is considered to study the governance of platform CSR.

The applicability of differential games is reflected in their current applications. For example, a researcher has modeled a zero-sum differential game between drone pursuers and vehicle evaders to address border security [33]. Other researchers have used differential games to research supply chain problems [34], environmental problems [35, 36]. It can be seen that differential games are also used to study equilibrium problems [37, 38]. Differential games are also used to study the strategy selection problem of agents. For example, scholars such as Zhang have established a differential game model with energy system operators leading and each energy system following, and studied the pricing strategy of operators and the optimization strategy of system operation [39]. The researcher considers social responsibility in the innovation process and uses differential games to investigate the strategies of innovation subjects [40]. It can be seen from the above research that differential games have been applied in many disciplines, which is mainly solves continuous dynamic problems, optimal strategy selection, coordination, and equilibrium problems.

## Research gaps in existing research

As can be seen from the relevant research literature on CSR governance, a rich theoretical foundation has been formed regarding the connotation of social responsibility of platform corporations, the governance subject of CSR, and the governance mechanism of CSR. Existing research provides a theoretical basis for this paper. However, there are still the following areas for improvement: (1) Most of the current research related to the connotation of social responsibility of platform corporations is dominated by Internet platform corporations, and fewer studies are conducted on platform corporations transformed by traditional corporations. (2) Most of the current research perspectives on the governance of social responsibility of traditional corporations are based on the internal subjects of the corporations, such as shareholders and directors of the corporations, or the influence of the academic background, gender, and knowledge of the macro-social environment of these subjects is taken into account. In the new economic context, traditional corporations use digital platforms to expand their innovation ecosystem externally, and governance subjects also need to consider other social subjects outside. Therefore, we go beyond the existing studies that focus on the roles of government and business in governance as the primary research perspectives. The study focus on the role of users and the public for socially responsible governance.(3) Current methods for researching platform CSR are primarily empirical [41] research studies. In platform corporate social responsibility governance, such methods cannot characterize the relationship between governance subject, and it is not possible to characters the continuous change in the governance process over time.

Therefore, this paper studies the governance of social responsibility of platform corporations from the perspective of social subjects in the context of platform transformation of traditional corporations. The governance subjects are government departments, platform corporations, users, and the public. Then, four decision models are constructed: decentralized decision-making without public and user supervision, centralized decision-making without public and user supervision, decentralized decision-making with public and user supervision, centralized decision-making with public and user supervision. Comparing the results of the four models using simulation analyzes, further examining the impact of different subjects and relevant parameters on the governance process of social responsibility, and arriving at an optimal governance model for the social responsibility of platform corporations. Finally, suggestions are provided for strategic choices of social responsibility governance for platform corporations.

The main contributions and innovations of this paper are summarized as follows: (1) Extracting the stakeholders of platform corporation social responsibility governance from the perspective of social subjects rather than the internal perspective of the corporation. This research perspective is more in line with the practical foundation of the platform innovation ecosystem. (2) Studying social responsibility governance process from the perspective of change over time allows us to study the dynamic change of the social responsibility governance process, which can better grasp the process laws of social responsibility governance (3) Using a mathematical model to describe the role of external monitoring on platform corporations’ social responsibility governance. In addition, each subject’s role in managing social responsibility deficiency and alienation is studied separately to provide a theoretical basis for the subject’s governance strategy.

## Problem description and research framework

Based on the summary of previous studies, the governance subjects of the lack of social responsibility and alienation of platform corporations are summarized as four, namely government departments, users, the government, and the public.

Firstly, the government is the primary actor in governance. Traditional corporations that have gone through platform transformation have more market resources, and corporations benefit from plentiful resources. They may use their dominant position to the detriment of others to maximize economic revenues. For example, they may stifle the growth of other corporations or monopolize the market. Because of the credibility of government legislation, the government can use legislation to regulate platform corporations’ lack of social responsibility and alienation [42, 43]. Platform corporations must abide by government regulations [44]. The government is the dominant player in platform CSR governance.

Secondly, another governor is the platform corporation itself. Platform corporations build platforms to generate revenue. so the ground rules for platform access are set by the platform corporations. Platform users, supply chain corporations, and partner organizations all have their interactions regulated by platform corporations. As a result, social responsibility is one of the elements of corporate governance [45], and corporations play an essential role in socially responsible governance. Based on research on the content and causes of corporate social responsibility, some researchers have proposed that self-discipline is one of measures to improve corporations’ enthusiasm to fulfill social responsibility.

The third aspect, monitoring by users and the public is indispensable. The lack of social responsibility and alienation of platform corporations must be governed by users and the general public. Users are the subjects who interact with resources on corporation platform, including users on the supply side of resources and users on the demand side of resources. The entry and exit mechanisms of enterprise platforms, as well as the standards of platform interaction, can affect user behavior. In addition, issues such as illegal access to industrial information [46], user data and information leakage, and substandard product quality have seriously harmed users’ interests. Users, therefore, need to monitor the behavior of platform corporations. The public is the social regulator of social responsibility, and public attitudes towards social responsibility can significantly impact compliance behavior. Whether it is government departments, platform users, or platform corporations, their socially responsible behaviors affect social welfare and social morality, which in turn affect the interests of the public. Thus, the public is concerned with societal interests and monitors the socially responsible behavior of other subjects.

**Fig 2:**
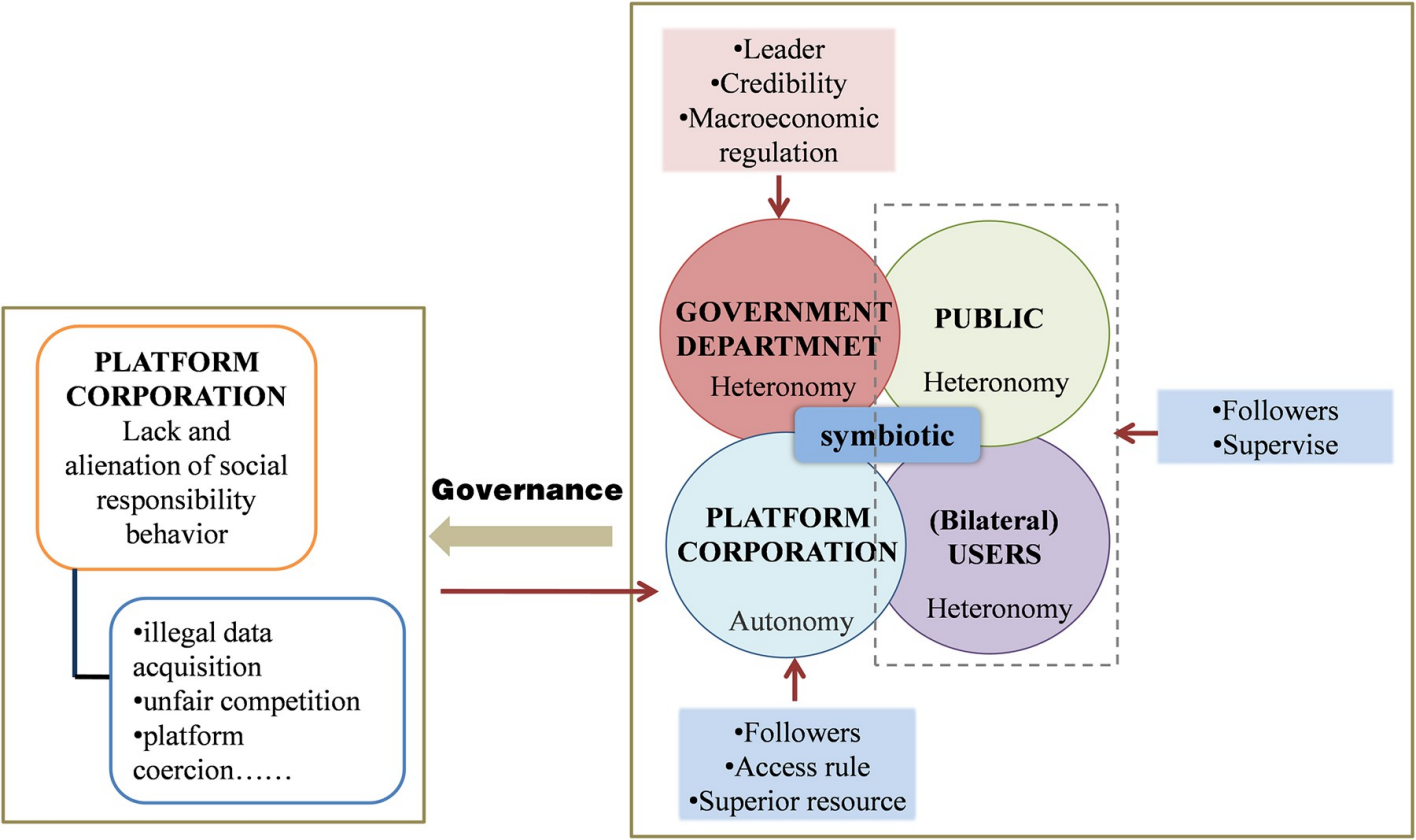
Research framework.

Based on the preceding analysis, the government and platform corporations are the governance subjects, and public and user monitoring are incorporated into the governance process, in which government agencies dominate, and platform corporations, the public, and users follow [47–49]. We have derived four decision models: decentralized decision-making without public and user supervision, centralized decision-making without public and user supervision, decentralized decision-making with public and user supervision, and centralized decision-making with public and user supervision. This model focuses on the following issues: First, what is the optimal trajectory of platform CSR governance volume and revenue? How does public and user, as well as government and platform corporations, affect the governance volume and revenue of platform CSR? Third, which will have a greater impact on CSR governance: public or user scrutiny? The research framework is shown in Fig 2.

## Model construction

This paper examines the governance of social responsibility in two contexts, whether or not public and user scrutiny is considered, by looking at the subjects of utility in the governance of social responsibility. Government agencies have an important role in the platform’s CSR governance. Government agencies encourage platform corporations to undertake governance by sharing the cost of corporate governance. At the same time, corporate social responsibility governance on the platform will be influenced by external public and user supervision, thus government agencies will provide incentives for it.

**Hypothesis 1.** Because governance efforts and cooperative innovation efforts [40] have comparable qualities in terms of determining the cost of the subject’s efforts, the cost function of the platform corporations can be expressed as:

$$C_{c}(E_{c}(t))=\frac{1}{2}k_{c}E_{c}^{2}(t)$$ (1)

The cost of governance for the government agencies is expressed as:

$$C_{g}(E_{g}(t))=\frac{1}{2}k_{g}E_{g}^{2}(t)$$ (2)

**Hypothesis 2.** Assuming that platform corporations and government agencies have a positive impact on the governance volume, and there is a natural decay rate for the governance volume, the differential equation for the change process of the governance volume is expressed as:

$$G^{'}(t)=cE_{c}(t)+gE_{g}(t)-\delta G(t)$$ (3)

*c* represents the effect coefficient of the government agencies effort degree of platform corporations on the change in governance volume, *g* represents the effect coefficient of the incentive effort degree of government agencies on the change of governance volume, and *δ* represents the natural attenuation rate of governance volume.

**Hypothesis 3.** Assuming that the revenue of governance is influenced by the governance volume, the revenue of governance is expressed as:

$$P(t)=P_{0}+\omega G$$ (4)

**Hypothesis 4.** The government subsidizes each governance subject at a time t to provide incentives. Assume that α, β, and γ denote the proportion of user and public supervision costs and the proportion of corporate governance costs, *α*< 1, *β*< 1, *γ*< 1. Assume that the discount rate for each subject is *r* (*r*> 0). The rates of revenue to the platform corporations, the public, the users and the government are assumed to be *τ*_*c*_, *τ*_*s*_, *τ*_*u*_ and *τ*_*g*_, respectively, and all are greater than zero.

### Decentralized decision-making without consideration of users and public scrutiny

Because the government department is the dominant player in platform CSR, it is the first to determine on the degree of governance effort when making decisions. Platform corporations decide their own optimal degree of governance efforts based on the decisions of government agencies. In a decentralized decision-making process that excludes public and user supervision, the government and the platform corporation each conduct their own governance and seek their own revenue maximization, where the optimal strategy for the government and the platform corporation are:

$$\gamma^{m}=\frac{2\tau_{g}-\tau_{c}}{2\tau_{g}+\tau_{c}}{E_{c}}^{m}=\frac{c\omega (2\tau_{g}+\tau_{c})}{2(r+\delta )k_{c}}{E_{g}}^{m}=\frac{g\tau_{g}\omega }{k_{g}(r+\delta )}$$ (5)

The optimal governance revenue for platform corporations and government agencies are, respectively are as following:

$${J_{C}}^{m}=e^{-rt}({D_{1}}^{m}G+{F_{1}}^{m}){J_{g}}^{m}=e^{-rt}({D_{2}}^{m}G+{F_{2}}^{m})$$ (6)

Where *D*_*1*_^*m*^, *D*_*2*_^*m*^, *F*_*1*_^*m*^, *F*_*2*_^*m*^ are as shown in Eq (16).

The optimal trajectory of the governance volume is:

$$G^{m}(t)=(G_{0}-H)e^{-\delta t}+H$$ (7)

Where $H=\frac{c^{2}\omega (2\tau_{g}+\tau_{c})}{2k_{c}(r+\delta )}+\frac{g^{2}\tau_{g}\omega }{k_{g}(r+\delta )}$

**Proof.** The governance revenue of platform corporations and government agencies are as following:

$$J_{c}(G,t)=\int_{0}^{\infty }e^{-rt}[\tau_{c}(P_{0}+\omega G)-\frac{\left(1-\gamma \right)}{2}k_{c}E_{c}^{2}]$$ (8)

$$J_{g}(G,t)=\int_{0}^{\infty }e^{-rt}[\tau_{g}(P_{0}+\omega G)-\frac{1}{2}k_{g}E_{g}^{2}-\frac{\gamma }{2}k_{c}E_{c}^{2}]$$ (9)

Using the dynamic stochastic control method, the following HJB equation is satisfied at moment t for the optimal revenue function of platform corporations, users, the public, and government agencies:

$$rV_{c}(G)=\underset{E_{c}\geq 0}{\max}[\tau_{c}(P_{0}+\omega G)-\frac{\left(1-\gamma \right)}{2}k_{c}E_{c}^{2}+{V_{c}}^{'}(cE_{c}+gE_{g}-\delta G(t))]$$ (10)

$$rV_{g}(G)=\underset{E_{g}\geq 0}{\max}[\tau_{g}(P_{0}+\omega G)-\frac{1}{2}k_{g}E_{g}^{2}-\frac{\gamma }{2}k_{c}E_{c}^{2}+{V_{g}}^{'}(cE_{c}+gE_{g}-\delta G(t))]$$ (11)

The optimal incentive coefficients for platform corporations as well as government agencies are determined from the first-order maximum condition, the optimal incentive coefficients can be obtained:

$$E_{c}=\frac{cV_{c}^{'}(G)}{(1-\gamma )k_{c}}E_{g}=\frac{gV_{g}^{'}(G)}{k_{g}}\gamma =\frac{2V_{g}^{'}-V_{c}^{'}}{2V_{g}^{'}+V_{c}^{'}}$$ (12)

Substitute Eq (12) into Eqs (10) and (11), the result is:

$$rV_{c}(G)=[\tau_{C}(P_{0}+\omega G)-\frac{\left(1-\gamma \right)}{2}k_{c}\frac{c^{2}V_{c}^{'2}}{{k_{c}}^{2}}]+V_{c}^{'}(\frac{c^{2}V_{c}^{'}}{k_{c}}+\frac{g^{2}V_{g}^{'}}{k_{g}}-\delta G)$$ (13)

$$rV_{g}(G)=[\tau_{g}(P_{0}+\omega G)-\frac{1}{2}k_{g}\frac{g^{2}V_{g}^{'2}}{{k_{g}}^{2}}-\frac{\gamma }{2}k_{c}\frac{c^{2}V_{c}^{'2}}{{k_{c}}^{2}}]+V_{g}^{'}(\frac{c^{2}V_{c}^{'}}{k_{c}}+\frac{g^{2}V_{g}^{'}}{k_{g}}-\delta G)$$ (14)

Based on the features of the differential equation in Eqs (13) and (14), it is assumed that a linear function about *G* is a solution of the HJB equation, and the variant formulas are as follows:

$$V_{c}(G)=D_{1}G+F_{1}V_{g}(G)=D_{2}G+F_{2}$$ (15)

Substitute Eq (15) into the Eqs (13) and (14), the result is:

$${D_{1}}^{m}=\frac{\tau_{c}\omega }{r+\delta }{D_{4}}^{m}=\frac{\tau_{g}\omega }{r+\delta }$$

$${F_{1}}^{m}=\frac{2a_{2}a_{1}^{2}c^{2}}{k_{c}r(2a_{2}+a_{1})}+\frac{a_{1}a_{2}g^{2}}{k_{g}r}+\frac{\tau_{c}P_{0}}{r}{F_{2}}^{m}=\frac{g^{2}a_{2}^{2}}{2k_{g}r}+\frac{c^{2}a_{1}(4a_{2}^{2}+a_{1}^{2})}{2k_{c}r(2a_{2}+a_{1})}+\frac{\tau_{g}P_{0}}{r}$$ (16)

Substitute Eq (16) into Eq (12), we get the optimal balance strategy for the government and platform corporations and the government’s optimal cost-sharing ratio, as shown in Eq (5). Substitute Eq (16) into Eqs (8) and (9), we get the revenue for the government and platform corporations, as shown in Eq (6). Substitute Eq (5) into Eq (3), we get the optimal trajectory for the governance volume, as shown in Eq (7).

### Centralized decision-making without consideration of users and public scrutiny

The optimal balance strategy for the platform corporation and the government, without taking into account centralized decision-making with public and user supervision, where the government department and the platform corporation are treated as a whole and the two participate in cooperative governance with the goal of maximizing overall revenue is:

$${E_{c}}^{Wm}=\frac{(\tau_{c}+\tau_{g})c\omega }{k_{c}(r+\delta )}{E_{g}}^{Wm}=\frac{(\tau_{c}+\tau_{g})g\omega }{k_{g}(r+\delta )}$$ (17)

The optimal governance revenue for all governance subjects is as flowing:

$$J^{Wm}=e^{-rt}(A_{1}G+B_{1})$$ (18)

Where *A*_1_ and *B*_1_are as shown in Eq (18).

Substitute Eq (30) into the Eq (4), the optimal trajectory for the governance volume is:

$$G^{Wm}(t)=(G_{0}-H^{W})e^{-\delta t}+H^{W}$$ (19)

Where $H^{W}=\frac{(\tau_{c}+\tau_{g})c^{2}\omega }{k_{c}(r+\delta )}+\frac{(\tau_{c}+\tau_{g})g^{2}\omega }{k_{g}(r+\delta )}$

**Proof.** The total governance revenue to the governance subjects of centralized decision-making without consideration of public and user oversight is as following:

$$J^{W}(G,t)=\int_{0}^{\infty }e^{-rt}[(\tau_{c}+\tau_{g})(P_{0}+\omega G)-\frac{1}{2}k_{g}E_{g}^{2}-\frac{1}{2}k_{c}E_{c}^{2}]$$ (20)

Using the dynamic stochastic control method, the following HJB equation is satisfied at moment t for the optimal function of total governance revenue:

$$rV^{W}(G)=\underset{E_{c},E_{b},E_{s},E_{g}}{\max}[\left(\tau_{c}+\tau_{g})(P_{0}+\omega G)-\frac{1}{2}k_{c}{E_{c}}^{2}-\frac{1}{2}k_{g}{E_{g}}^{2}+V^{W'}(G)(\begin{array}{l} cE_{c}(t)\\ +gE_{g}(t)-\delta G(t) \end{array}\right)]$$ (21)

The optimal incentive coefficients for platform corporations as well as government agencies are determined from the first-order maximum condition; the optimal incentive coefficients can be obtained:

$${E_{c}}^{W}=\frac{cV^{W'}(G)}{k_{c}}{E_{g}}^{W}=\frac{gV^{W'}(G)}{k_{g}}$$ (22)

Substitute Eq (22) into the Eq (21), the following equation can be obtained:

$$rV^{W}(G)=(\tau_{c}+\tau_{g})(P_{0}+\omega G)-\frac{1}{2}k_{c}\frac{c^{2}V^{W^{'2}}}{{k_{c}}^{2}}-\frac{1}{2}k_{g}\frac{g^{2}V^{W^{'2}}}{{k_{g}}^{2}}+V^{W^{'}}(\frac{c^{2}V^{W^{'}}}{k_{c}}+\frac{g^{2}V^{W^{'}}}{k_{g}}-\delta G)$$ (23)

Based on the features of the differential equation in Eq (23), it is assumed that a linear function about *G* is a solution of the HJB equation, and the variant formulas are as follows:

$$V^{W}(G)=A_{1}G+B_{1}$$ (24)

Substitute Eq (24) into the Eq (23), the following equation can be obtained:

$$A_{1}=\frac{(\tau_{c}+\tau_{g})\omega }{r+\delta }B_{1}=\frac{c^{2}{A_{1}}^{2}}{2rk_{c}}+\frac{g^{2}{A_{1}}^{2}}{2rk_{g}}+\frac{(\tau_{c}+\tau_{g})P_{0}}{r}$$ (25)

Substitute Eq (25) into Eq (22), we get the optimal balance strategy for the government and platform corporations, as shown in Eq (17). Substitute Eq (25) into Eq (20), we get the revenue for the government and platform corporations, as shown in Eq (18). Substitute Eq (17) into Eq (3), we get the optimal trajectory for the governance volume, as shown in Eq (19).

### Decentralized decision-making with consideration of users and public scrutiny

Decentralized decision-making that considers public and user supervision is one that incorporates public and user examination into the governance process. Governing subjects want to maximize their own advantages. Suppose that the price of public and user oversight is expressed as:

$$C_{s}(E_{s}(t))=\frac{1}{2}k_{s}E_{s}^{2}(t)C_{u}(E_{u}(t))=\frac{1}{2}k_{u}E_{u}^{2}(t)$$ (26)

Assuming that more public and user supervision will increase the benefits of government. Taking the literature [50] on the impact of preferences into account, the revenue of governance is represented as flowing:

$$P(t)=P_{0}+sE_{s}(t)+uE_{u}(t)+z\omega G$$ (27)

Where s and u represents the effort coefficient of public and user supervision on governance revenue (*s*, *u*>0), and *z* represents the consciousness coefficient of public and user supervision on governance revenue (*z*>1).

Considering public and user supervision, the optimal balance strategy for every governance subject and optimal incentive coefficients of government agencies are as following:

$${E_{c}}^{Nm}=\frac{c\omega z(2\tau_{g}+\tau_{c})}{2(r+\delta )k_{c}}{E_{s}}^{Nm}=\frac{s(2\tau_{g}+\tau_{s})}{2k_{s}}{E_{u}}^{Nm}=\frac{u(2\tau_{g}+\tau_{u})}{2k_{u}}{E_{g}}^{Nm}=\frac{g\tau_{g}\omega z}{k_{g}(r+\delta )}$$

$$\gamma^{m}=\frac{2\tau_{g}-\tau_{c}}{2\tau_{g}+\tau_{c}}\beta^{m}=\frac{2\tau_{g}-\tau_{s}}{2\tau_{g}+\tau_{s}}\alpha^{m}=\frac{2\tau_{g}-\tau_{u}}{2\tau_{g}+\tau_{u}}$$ (28)

The optimal governance revenue for every governance subject is as following:

$${J_{C}}^{Nm}=e^{-rt}({d_{1}}^{m}G+{f_{1}}^{m}){J_{s}}^{Nm}=e^{-rt}({d_{2}}^{m}G+{f_{2}}^{m})$$

$${J_{u}}^{Nm}=e^{-rt}({d_{3}}^{m}G+{f_{3}}^{m}){J_{g}}^{Nm}=e^{-rt}({d_{4}}^{m}G+{f_{4}}^{m})$$ (29)

Where *d*_1_^m^, *d*_2_^m^, *d*_3m_, *d*_4_^m^, *f*_1_^m^, *f*_2_^m^, *f*_3_^m^, *f*_4_^m^ are as shown in Eq (45).

The optimal trajectory of the governance volume is:

$$G^{Nm}(t)=(G_{0}-H^{N})e^{-\delta t}+H^{N}$$ (30)

Where $H^{N}=\frac{c^{2}\omega z(2\tau_{g}+\tau_{c})}{2k_{c}(r+\delta )}+\frac{g^{2}\tau_{g}\omega z}{k_{g}(r+\delta )}$ **Proof.** The revenue of governance by government agencies, platform corporations, public, and user are as following:

$${J_{g}}^{N}(G,t)=\int_{0}^{\infty }e^{-rt}[\tau_{g}(P_{0}+sE_{s}(t)+uE_{u}(t)+z\omega G)-\frac{1}{2}k_{g}E_{g}^{2}-\frac{\gamma }{2}k_{c}E_{c}^{2}-\frac{\beta }{2}k_{s}E_{s}^{2}-\frac{\alpha }{2}k_{u}E_{u}^{2}]$$ (31)

$${J_{c}}^{N}(G,t)=\int_{0}^{\infty }e^{-rt}[\tau_{c}(P_{0}+sE_{s}(t)+uE_{u}(t)+z\omega G)-\frac{\left(1-\gamma \right)}{2}k_{c}E_{c}^{2}]$$ (32)

$${J_{s}}^{N}(G,t)=\int_{0}^{\infty }e^{-rt}[\tau_{s}(P_{0}+sE_{s}(t)+uE_{u}(t)+z\omega G)-\frac{\left(1-\beta \right)}{2}k_{s}E_{s}^{2}]$$ (33)

$${J_{u}}^{N}(G,t)=\int_{0}^{\infty }e^{-rt}[\tau_{u}(P_{0}+sE_{s}(t)+uE_{u}(t)+z\omega G)-\frac{\left(1-\alpha \right)}{2}k_{u}E_{u}^{2}]$$ (34)

Using the dynamic stochastic control method, the following HJB equation is satisfied at moment t for the optimal function of platform corporation revenue, users, the public, and government agencies:

$$r{V_{c}}^{N}(G)=\underset{E_{c}\geq 0}{\max}[\tau_{c}(P_{0}+sE_{s}(t)+uE_{u}(t)+z\omega G)-\frac{\left(1-\gamma \right)}{2}k_{c}E_{c}^{2}+{V_{c}}^{'}(cE_{c}+gE_{g}-\delta G(t))]$$ (35)

$$r{V_{s}}^{N}(G)=\underset{E_{s}\geq 0}{\max}[\tau_{s}(P_{0}+sE_{s}(t)+uE_{u}(t)+z\omega G)-\frac{\left(1-\beta \right)}{2}k_{s}E_{s}^{2}+{V_{s}}^{'}(cE_{c}+gE_{g}-\delta G(t))]$$ (36)

$$r{V_{u}}^{N}(G)=\underset{E_{u}\geq 0}{\max}[\tau_{u}(P_{0}+sE_{s}(t)+uE_{u}(t)+z\omega G)-\frac{\left(1-\alpha \right)}{2}k_{u}E_{u}^{2}+{V_{u}}^{'}(cE_{c}+gE_{g}-\delta G(t))]$$ (37)

$$r{V_{g}}^{N}(G)=\underset{E_{g}\geq 0}{\max}[\begin{array}{l} \tau_{g}(P_{0}+sE_{s}(t)+uE_{u}(t)+z\omega G)-\frac{1}{2}k_{g}E_{g}^{2}-\frac{\gamma }{2}k_{c}E_{c}^{2}\\ -\frac{\beta }{2}k_{s}E_{s}^{2}-\frac{\alpha }{2}k_{u}E_{u}^{2}+{V_{g}}^{'}(cE_{c}+gE_{g}-\delta G(t)) \end{array}]$$ (38)

The optimal incentive coefficients for platform corporations, the public, users, and government agencies are determined from the first-order maximum condition, the optimal incentive coefficients can be obtained:

$${E_{c}}^{N}=\frac{cV_{c}^{'}(G)}{(1-\gamma )k_{c}}{E_{s}}^{N}=\frac{s\tau_{s}}{(1-\beta )k_{s}}{E_{u}}^{N}=\frac{u\tau_{u}}{(1-\alpha )k_{u}}{E_{g}}^{N}=\frac{gV_{g}^{'}(G)}{k_{g}}$$

$$\gamma =\frac{2V_{g}^{'}-V_{c}^{'}}{2V_{g}^{'}+V_{c}^{'}}\beta =\frac{2\tau_{g}-\tau_{s}}{2\tau_{g}+\tau_{s}}\alpha =\frac{2\tau_{g}-\tau_{u}}{2\tau_{g}+\tau_{u}}$$ (39)

Substitute Eq (39) into the Eqs (35)–(38), the following equation can be obtained:

$$r{V_{c}}^{N}(G)=[\tau_{C}(P_{0}+s\frac{\tau_{s}s}{k_{s}}+u\frac{\tau_{u}u}{k_{u}}+z\omega G)-\frac{\left(1-\gamma \right)}{2}k_{c}\frac{c^{2}V_{c}^{'2}}{{k_{c}}^{2}}]+V_{c}^{'}(\frac{c^{2}V_{c}^{'}}{k_{c}}+\frac{g^{2}V_{g}^{'}}{k_{g}}-\delta G)$$ (40)

$$r{V_{s}}^{N}(G)=[\tau_{s}(P_{0}+s\frac{\tau_{s}s}{k_{s}}+u\frac{\tau_{u}u}{k_{u}}+z\omega G)-\frac{\left(1-\beta \right)}{2}k_{s}\frac{s^{2}{\tau_{s}}^{2}}{{k_{s}}^{2}}]+V_{s}^{'}(\frac{c^{2}V_{c}^{'}}{k_{c}}+\frac{g^{2}V_{g}^{'}}{k_{g}}-\delta G)$$ (41)

$$r{V_{u}}^{N}(G)=[\tau_{u}(P_{0}+s\frac{\tau_{s}s}{k_{s}}+u\frac{\tau_{u}u}{k_{u}}+z\omega G)-\frac{\left(1-\alpha \right)}{2}k_{u}\frac{u^{2}{\tau_{u}}^{2}}{{k_{u}}^{2}}]+V_{u}^{'}(\frac{c^{2}V_{c}^{'}}{k_{c}}+\frac{g^{2}V_{g}^{'}}{k_{g}}-\delta G)$$ (42)

$$r{V_{g}}^{N}(G)=[\tau_{g}(P_{0}+s\frac{\tau_{s}s}{k_{s}}+u\frac{\tau_{u}u}{k_{u}}+z\omega G)-\frac{1}{2}k_{g}\frac{g^{2}V_{g}^{'2}}{{k_{g}}^{2}}-\frac{\gamma }{2}k_{c}\frac{c^{2}V_{c}^{'2}}{{k_{c}}^{2}}-\frac{\beta }{2}k_{s}\frac{s^{2}{\tau_{s}}^{2}}{{k_{s}}^{2}}-\frac{\alpha }{2}k_{u}\frac{u^{2}{\tau_{u}}^{2}}{{k_{u}}^{2}}]+V_{g}^{'}(\frac{c^{2}V_{c}^{'}}{k_{c}}+\frac{g^{2}V_{g}^{'}}{k_{g}}-\delta G)$$ (43)

Based on the features of the differential equation in Eqs (40)–(43), it is assumed that a linear function about *G* is a solution of the HJB equation, and the variant formulas are as follows:

$${V_{c}}^{N}(G)=d_{1}G+f_{1}{V_{s}}^{N}(G)=d_{2}G+f_{2}{V_{u}}^{N}(G)=d_{3}G+f_{3}{V_{g}}^{N}(G)=d_{4}G+f_{4}$$ (44)

Substitute Eq (44) into the Eqs (40)–(43), the following equation can be obtained:

$${d_{1}}^{m}=\frac{\tau_{c}\omega z}{r+\delta }{d_{2}}^{m}=\frac{\tau_{s}\omega z}{r+\delta }{d_{3}}^{m}=\frac{\tau_{u}\omega z}{r+\delta }{d_{4}}^{m}=\frac{\tau_{g}\omega z}{r+\delta }$$

$${f_{1}}^{m}=\frac{s^{2}\tau_{c}\tau_{s}}{rk_{s}}+\frac{u^{2}\tau_{c}\tau_{u}}{rk_{u}}+\frac{g^{2}d_{1}d_{4}}{rk_{g}}+\frac{2c^{2}{d_{1}}^{2}d_{4}}{rk_{c}(2d_{4}+d_{1})}+\frac{\tau_{c}P_{0}}{r}$$

$${f_{2}}^{m}=\frac{u^{2}\tau_{s}\tau_{u}}{rk_{u}}+\frac{c^{2}d_{1}d_{2}}{rk_{c}}+\frac{g^{2}d_{2}d_{4}}{rk_{g}}+\frac{2s^{2}{\tau_{s}}^{2}\tau_{g}}{rk_{s}(2\tau_{g}+\tau_{s})}+\frac{\tau_{s}P_{0}}{r}$$

$${f_{3}}^{m}=\frac{s^{2}\tau_{s}\tau_{u}}{rk_{s}}+\frac{c^{2}d_{1}d_{3}}{rk_{c}}+\frac{g^{2}d_{3}d_{4}}{rk_{g}}+\frac{2u^{2}{\tau_{u}}^{2}\tau_{g}}{rk_{u}(2\tau_{g}+\tau_{u})}+\frac{\tau_{u}P_{0}}{r}$$

$${f_{4}}^{m}=\frac{g^{2}{d_{4}}^{2}}{2k_{g}r}+\frac{s^{2}\tau_{s}(4{\tau_{g}}^{2}+{\tau_{s}}^{2})}{2k_{s}(2\tau_{g}+\tau_{s})r}+\frac{u^{2}\tau_{u}(4{\tau_{g}}^{2}+{\tau_{u}}^{2})}{2k_{u}(2\tau_{g}+\tau_{u})r}+\frac{c^{2}d_{1}(4{d_{4}}^{2}+{d_{1}}^{2})}{2k_{c}(2d_{4}+d_{1})r}+\frac{\tau_{g}P_{0}}{r}$$ (45)

Substitute Eq (45) into Eq (39), we get the optimal balance strategy for every governance subjects and optimal incentive coefficients of government agencie, as shown in Eq (28). Substitute Eq (45) into Eqs (31)–(34), we get the revenue for every governance subject, as shown in Eq (18). Substitute Eq (17) into Eq (3), we get the optimal trajectory for the governance volume, as shown in Eq (19).

### Centralized decision-making with consideration of users and public scrutiny

The optimal balance strategy for all governance subjects, taking into consideration centralized decision-making with public and user supervision, where all governance subjects are viewed as a whole and all participate in cooperative governance to maximize overall revenue, is:

$${E_{c}}^{WTm}=\frac{(\tau_{c}+\tau_{s}+\tau_{u}+\tau_{g})c\omega z}{k_{c}(r+\delta )}{E_{s}}^{WTm}=\frac{(\tau_{c}+\tau_{s}+\tau_{u}+\tau_{g})s}{k_{s}}$$

$${E_{u}}^{WTm}=\frac{(\tau_{c}+\tau_{s}+\tau_{u}+\tau_{g})u}{k_{u}}{E_{g}}^{WTm}=\frac{(\tau_{c}+\tau_{s}+\tau_{u}+\tau_{g})g\omega z}{k_{g}(r+\delta )}$$ (46)

The optimal governance revenue for all governance subjects is as following:

$$J^{WTm}=e^{-rt}(a_{1}G+b_{1})$$ (47)

Where*a*_1_ and *b*_1_are as shown in Eq (54).

The optimal trajectory of the governance volume is:

$$G^{WTm}(t)=(G_{0}-H^{WT})e^{-\delta t}+H^{WT}$$ (48)

Where $H^{WT}=\frac{(\tau_{c}+\tau_{s}+\tau_{u}+\tau_{g})c^{2}\omega z}{k_{c}(r+\delta )}+\frac{(\tau_{c}+\tau_{s}+\tau_{u}+\tau_{g})g^{2}\omega z}{k_{g}(r+\delta )}$

**Proof.** The total revenue to platform corporations, users, the public and government agencies is:

$$J^{WT}(G,t)=\int_{0}^{\infty }e^{-rt}[(\tau_{c}+\tau_{s}+\tau_{u}+\tau_{g})(P_{0}+sE_{S}+uE_{u}+z\omega G)-\frac{1}{2}k_{g}E_{g}^{2}-\frac{1}{2}k_{c}E_{c}^{2}-\frac{1}{2}k_{s}E_{s}^{2}-\frac{1}{2}k_{u}E_{u}^{2}]$$ (49)

Using the dynamic stochastic control method, the following HJB equation is satisfied at moment *t* for the optimal function of total governance revenue:

$$rV^{WT}(G)=\underset{E_{c},E_{b},E_{s},E_{g}}{\max}[\left(\tau_{c}+\tau_{s}+\tau_{u}+\tau_{g})(P_{0}+sE_{S}+uE_{u}+z\omega G)-\frac{1}{2}k_{c}{E_{c}}^{2}-\frac{1}{2}k_{s}{E_{s}}^{2}-\frac{1}{2}k_{u}{E_{u}}^{2}-\frac{1}{2}k_{g}{E_{g}}^{2}+V^{WT'}(G)(cE_{c}(t)+gE_{g}(t)-\delta G(t)\right)]$$ (50)

The optimal incentive coefficients for platform corporations, the public, users, and government agencies are determined from the first-order maximum condition, the optimal incentive coefficients can be obtained:

$${E_{c}}^{WT}=\frac{cV^{WT'}(G)}{k_{c}}{E_{s}}^{WT}=\frac{(\tau_{c}+\tau_{s}+\tau_{u}+\tau_{g})s}{k_{s}}{E_{u}}^{WT}=\frac{(\tau_{c}+\tau_{s}+\tau_{u}+\tau_{g})u}{k_{u}}{E_{g}}^{WT}=\frac{gV^{WT'}(G)}{k_{g}}$$ (51)

Substitute Eq (51) into the Eq (50), the following equation can be obtained:

$$\begin{array}{l} rV^{WT}(G)=(\tau_{c}+\tau_{s}+\tau_{u}+\tau_{g})(P_{0}+\frac{(\tau_{c}+\tau_{s}+\tau_{u}+\tau_{g})s^{2}}{k_{s}}+\frac{(\tau_{c}+\tau_{s}+\tau_{u}+\tau_{g})u^{2}}{k_{u}}+\omega zG)-\frac{1}{2}k_{c}\frac{c^{2}V^{WT'2}}{{k_{c}}^{2}}\\ \frac{1}{2}k_{u}\frac{{\left(\tau_{c}+\tau_{s}+\tau_{u}+\tau_{g}\right)}^{2}u^{2}}{{k_{u}}^{2}}-\frac{1}{2}k_{s}\frac{{\left(\tau_{c}+\tau_{s}+\tau_{u}+\tau_{g}\right)}^{2}s^{2}}{{k_{s}}^{2}}-\frac{1}{2}k_{g}\frac{g^{2}V^{WT'2}}{{k_{g}}^{2}}+V^{WT'}(\frac{c^{2}V^{WT'}}{k_{c}}+\frac{g^{2}V^{WT'}}{k_{g}}-\delta G) \end{array}$$ (52)

Based on the features of the differential equation in Eq (52), it is assumed that a linear function about G is a solution of the HJB equation such that:

$$V^{WT}(G)=a_{1}G+b_{1}$$ (53)

Substitute Eq (53) into the Eq (52), the following equation can be obtained:

$$a_{1}=\frac{(\tau_{c}+\tau_{s}+\tau_{u}+\tau_{g})\omega z}{r+\delta }$$

$$b_{1}=\frac{{\left(\tau_{c}+\tau_{s}+\tau_{u}+\tau_{g}\right)}^{2}s^{2}}{2rk_{s}}+\frac{{\left(\tau_{c}+\tau_{s}+\tau_{u}+\tau_{g}\right)}^{2}u^{2}}{2rk_{u}}+\frac{c^{2}{a_{1}}^{2}}{2rk_{c}}+\frac{g^{2}{a_{1}}^{2}}{2rk_{g}}+\frac{(\tau_{c}+\tau_{s}+\tau_{u}+\tau_{g})P_{0}}{r}$$ (54)

Substitute Eq (54) into Eq (51), we get the optimal balance strategy for every governance subject, as shown in Eq (46). Substitute Eq (54) into Eq (49), we get the optimal governance revenue for all governance subjects, as shown in Eq (47). Substitute Eq (46) into Eq (3), we get the optimal trajectory for the governance volume, as shown in Eq (48).

## Comparative analysis and numerical simulation

### Comparative analysis

Corollary 1 and 2 can be obtained by comparing the respective optimal strategies and optimal revenue of platform corporations, governance subjects, users and public under the four game modes.

**Corollary 1.** The following corollary can be drawn from a comparison of situations where public and user supervision is not taken into account and when public and users are taken into consideration.


$$\Delta J_{1}=J^{Nm}-J^{m}\geq 0\Delta J_{2}=J^{WTm}-J^{Wm}\geq 0$$

$$\Delta G_{1}=G^{Nm}-G^{m}\geq 0\Delta G_{2}=G^{WTm}-G^{Wm}\geq 0$$

$$\Delta E_{c1}={E_{c}}^{Nm}-{E_{c}}^{m}\geq 0\Delta E_{c2}={E_{c}}^{WTm}-{E_{c}}^{Wm}\geq 0$$

$$\Delta E_{g1}={E_{g}}^{Nm}-{E_{g}}^{m}\geq 0\Delta E_{g2}={E_{g}}^{WTm}-{E_{g}}^{Wm}\geq 0$$ (55)

The corollary proved that when public and user supervision is considered, governance volume and governance revenue are higher than when no supervision is considered, which demonstrates that public and user supervision can boost governance’s revenue and reduce relationship alienation. The level of optimal governance efforts of platform corporations and government agencies rises when the public and users are taken into account. Therefore, platform corporations and government agencies will exert more effort regarding platform CSR governance when the public and users are considered.

**Corollary 2.** The government departments’ optimal level of governance effort and platform corporations’ optimal level of governance effort is proportional to the effect coefficient of the effort level on the governance volume, the effect coefficient of the governance volume on the governance revenue, and the respective rates of return. In addition, the platform corporations’ optimal level of governance effort is also proportional to the government’s incentive coefficient. The platform corporations’ optimal level of governance effort is inversely proportional to the decay rate of the governance volume and cost coefficient.

In the case of considering the supervision of the public and users, public and users’ optimal level of governance effort is proportional to the effect coefficient of the effort level on the governance volume and rates of return. The public and users’ optimal level of governance effort is inversely proportional to the cost coefficient. In decentralized decision-making, the public and users’ optimal level of supervision effort is also proportional to the government’s incentive coefficient.

Corollary 2 shows that the higher the effect of government departments and platform corporations’ governance volume on revenue, the more perceptible the governance effort’s level on the governance volume, the more revenue each receives, and the higher of governance effort’s level.

The more significant the effect of the public and users’ level of supervision effort on revenue, the higher the respective supervision revenue, and the lower the supervision costs, the more motivated the public and users are to monitor social responsibility shortfalls and alienation.

### Numerical simulation

#### Data source

To further analyze the effect of different parameters on the amount and revenue of governance, the model was simulated using MATLAB2016a software. As this paper establishes the governance decision-making model of platform CSR based on the perspective of multiple subjects, it is not easy to obtain the exact data related to the government, corporations, users, and the public. On the other hand, referring to the relevant literature for the subject’s decision-making behavior and the related research on differential games, the final result can be reflected by self-assignment. Moreover, there have been studies using assignment methods to simulate and verify differential game models [51]. Therefore, this paper refers to the literature [52, 53] and combines the author’s previous research [54] to assign values to the parameters involved in this paper. The parameters were assigned the following values:

*c* = 4,s = 3,*u* = 3,g = 5,*k*_*c*_ = 20,*k*_*s*_ = 30,*k*_*u*_ = 12,*k*_*g*_ = 20,*r* = 0.5,*δ* = 0.6,*ω* = 3,*τ*_*c*_ = 0.3,*τ*_*s*_ = 0.2,*τ*_*u*_ = 0.2,*τ*_*g*_ = 0.3,*G*_*0*_ = 1,*P*_*0*_ = 1, *z* = 10. Subsequently, the model was programmed using simulation software to obtain dynamic curves of governance volume and governance revenue.

### Analysis of simulation result

To further analyze the influence of parameter changes on the model results, the model’s simulation results are analyzed. When performing the analysis, set the other parameters unchanged and change only the parameters to be studied.

**Fig 3:**
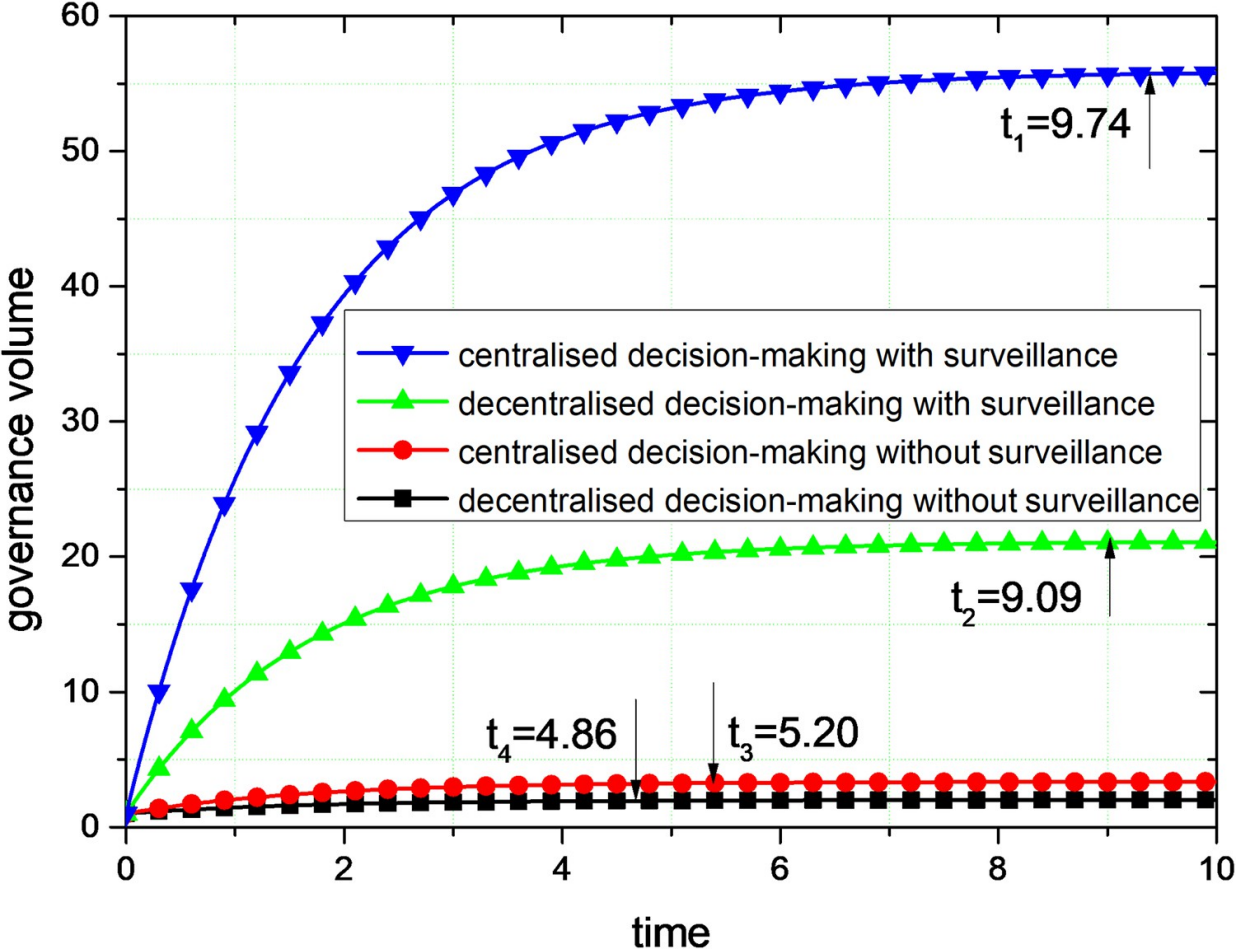
Comparison of the governance volume.

Fig 3 illustrates the trend of governance volume over time. With the increasing level of user and public supervision, the volume of governance is getting higher and reaching a stable value. Decentralized decision-making with public and user supervision has increased governance by 9.5 times. The centralized decision-making with public and user supervision has increased the amount of governance by 15.69 times. Whether or not considering user and public supervision, centralized decision-making can generate greater governance volume. In terms of time to reach stabilization, the unsupervised decentralized governance volume reaches stabilization at 4.86S. Supervised centralized governance takes the second place, and reaches stabilization at 5.20S. The time for achieving stability in supervised decentralized governance is ranked third, with 9.09s. Supervised centralized governance is the last to achieve stability with a stabilization time of 9.74s. In terms of the volume of governance, centralized decision-making takes longer to reach stability than decentralized decision-making.

The result shows that faced with the complex and changing external environment and the development of the sharing economy, the relationship between the public and users and platform corporations is closer. The joint supervision of the public and users can encourage platform corporations to perform social responsibility behaviors such as disclosing social responsibility information. Although centralized decision-making needs more time to balance the interests of various governance subjects, it generates a higher volume of governance.

**Fig 4:**
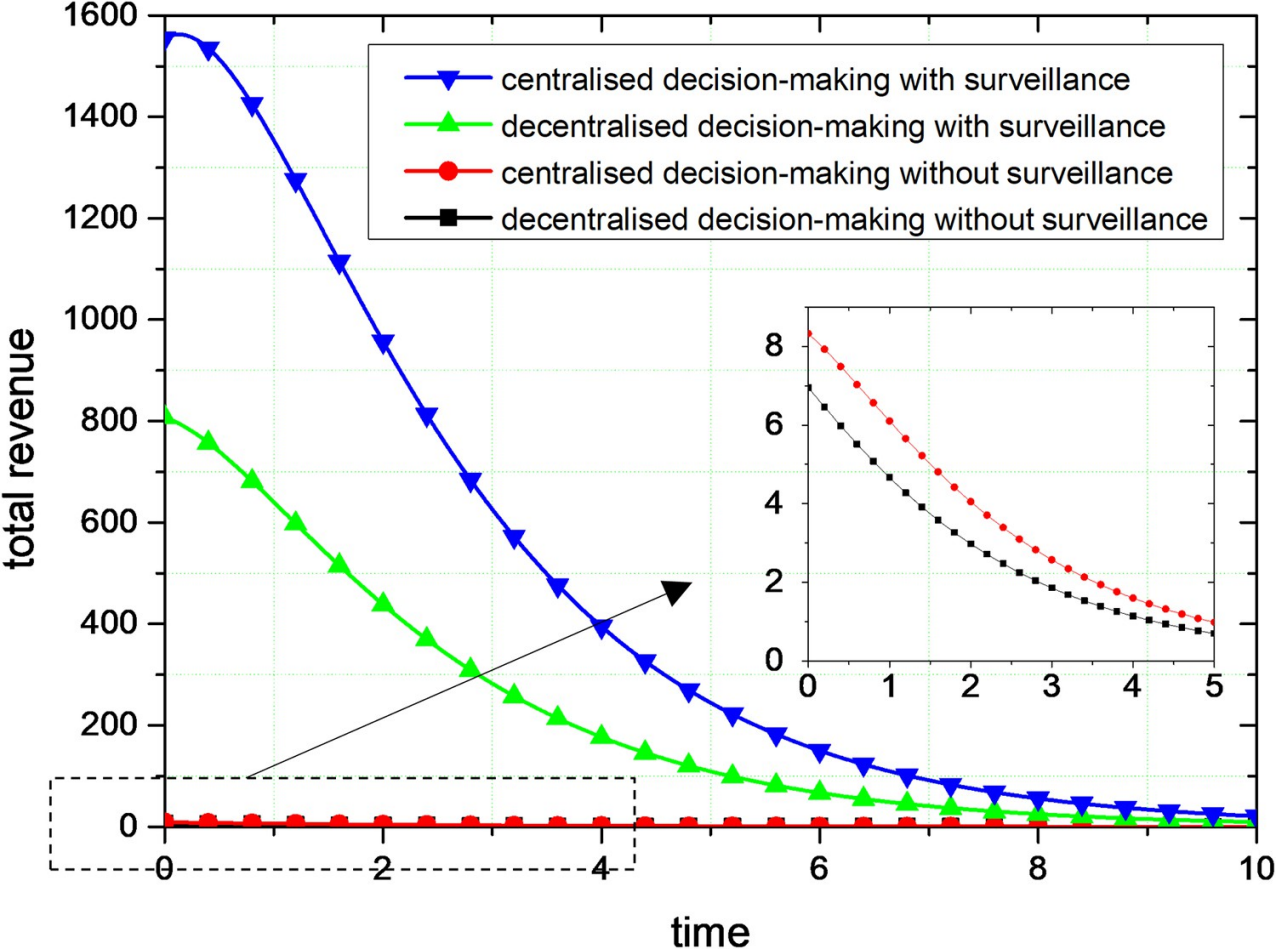
Comparison of governance revenue.

As shown in Fig 4, the total revenue of governance will drop over time and eventually stabilize. In conjunction with Figure, the total revenue declines as the volume of governance increases, until it reaches one revenue level. This phenomenon occurs because, as the amount of governance increases, the problem of social responsibility that can be governed reduces. The benefits of governance are lowest when social responsibility concerns are fully governed. This phenomenon also illustrates that social responsibility is the pursuit of social goals, and the presence of conflicts with commercial interests.

The governance of behaviours such as lack of social responsibility and alienation requires a significant cost, so a reasonable governance decision-making mechanism is needed to balance commercial interests and social benefits. The governance revenue is greater than zero, which is also a good illustration of the possible positive economic benefits.

By comparing the simulation results in Figs 3 and 4, centralized decision-making can get higher governance volume and governance revenue compared with decentralized decision-making. Therefore, for the lack of social responsibility and the alienation of social responsibility of platform corporations, the collaborative governance of multiple subjects is the optimal governance. In the process of social responsibility, multiple governance subjects form a unified whole, and the governance of social responsibility of platform corporations pursues the overall interests of this unity rather than maximizing individual interests. The magnitude of change in the governance volume and the revenue of governance are greater with centralized decision-making, suggesting that the governance effects of centralized decision-making are more readily apparent.

**Fig 5:**
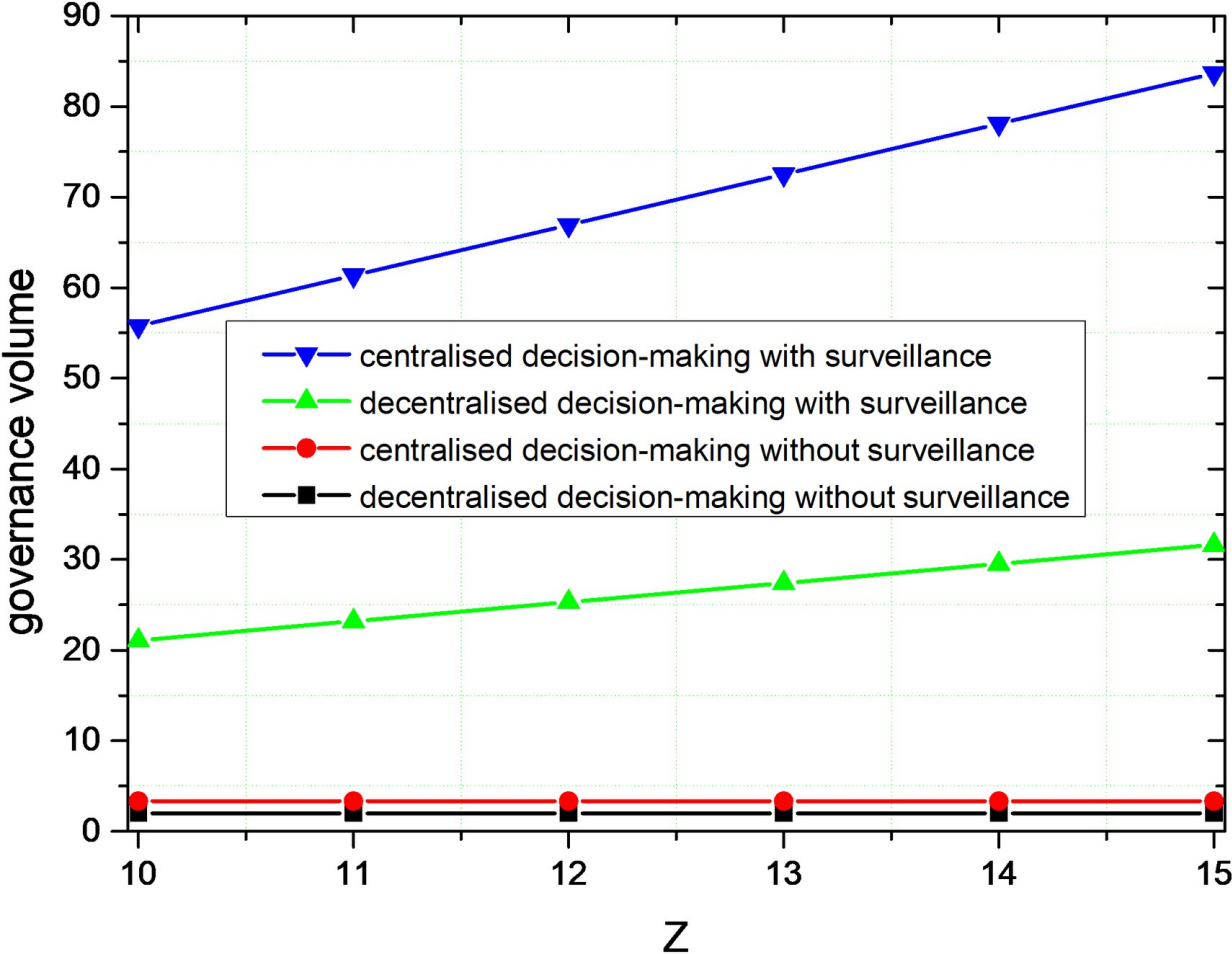
Effect of parameter z on the governance volume.

The effect of parameter *z* on the governance volume is shown in Fig 5. As seen in Fig 5, at the moment of *t* = 10, the volume of governance increases as the value of *z* increases, and the greater the awareness of public and user supervision, the greater of governance volume of social responsibility issues and the more influential the governance. As a result, in addition to government regulation, external scrutiny forces companies to fulfill their social responsibilities for the sake of their reputation and public pressure.

**Fig 6:**
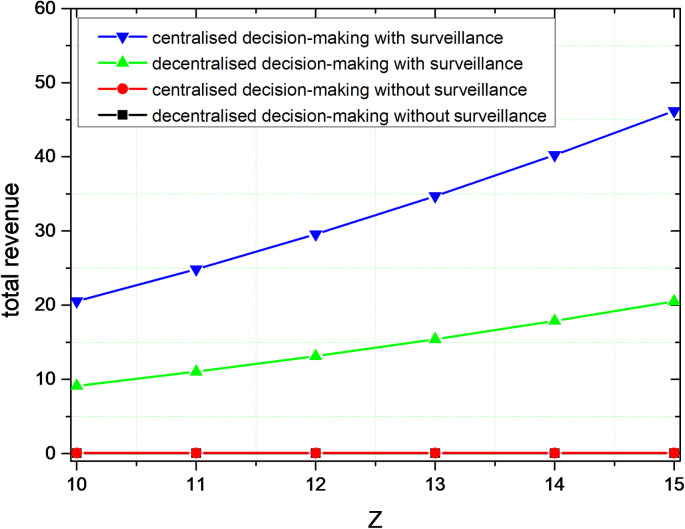
Effect of parameter *z* on the total revenue.

The effect of parameter *z* on governance revenue is depicted in Fig 6. Fig 6 shows that at *t* = 10, the total revenue rises as the value of *z* grows, which is that the overall revenue of governance will increase with greater public and user awareness.Because users rely on the enterprise’s platform to interact with resources, the formulation of platform rules is closely related to fair transactions between users. Users’ awareness of social responsibility supervision will promote corporate to solve social responsibility alienation and lack of social responsibility. The role of the public in the governance of platform CSR is manifested in the guidance of social opinion. The public can expose violations of the law through various media, and the stronger the public’s awareness of the platform’s corporate social responsibility, the more proactive it will be in monitoring violations of the law, thus contributing to the improvement of governance efficiency.

**Fig 7:**
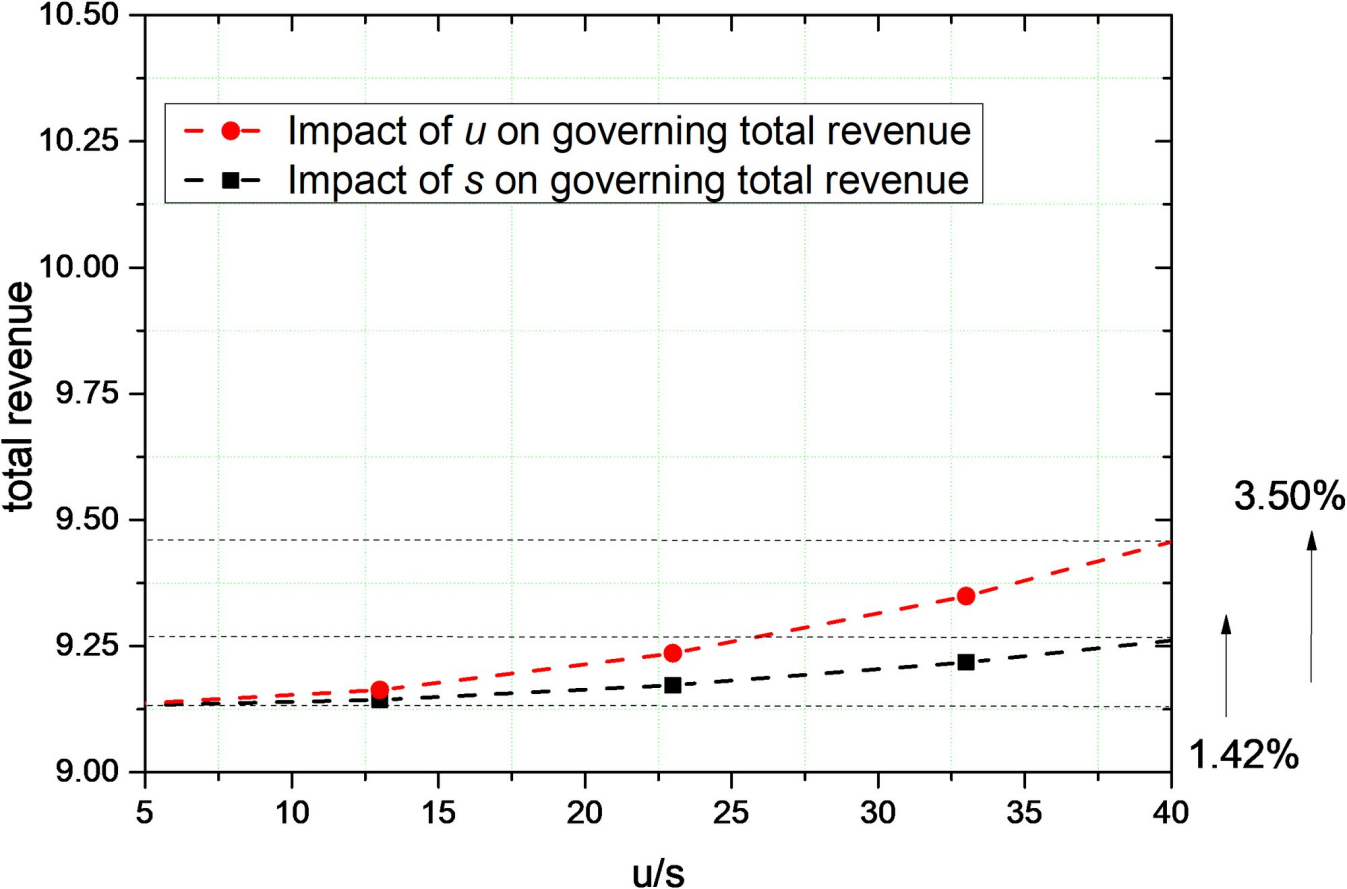
In decentralized governance that considers public and user supervision, the effect of changes in parameters *s* and *u* on the total governance revenue.

The effect of changes in the parameters *s* and *u* under decentralized decision-making with public and user supervision on total governance revenue is depicted in Fig 7. The curves show the trend of the effect of the public on the total revenue of governance and the trend of the effect of users on the total revenue of governance under decentralized decision-making. At the moment of *t* = 10, when the increasing amount of parameter *s* and parameter *u* is the same, the user’s supervision increases the governance revenue by 3.5%, and the public’s supervision increases the governance revenue by 1.42%. In the case of decentralized decision-making, user supervision has a more significant effect on the total revenue of governance.

**Fig 8:**
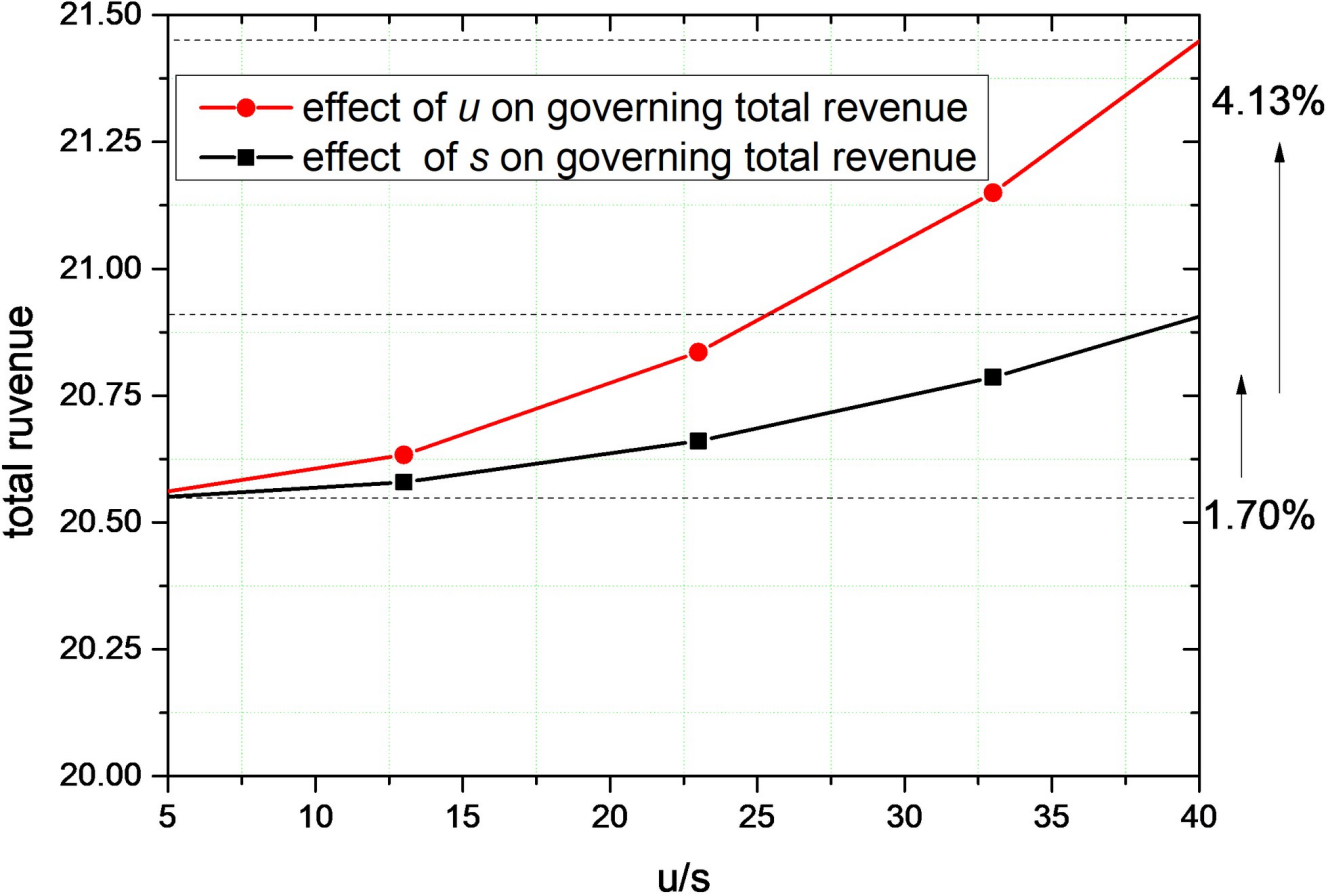
In centralized governance that considers public and user supervision, the effect of changes in parameters s and u on the total governance revenue.

The effect of changes in the parameters *s* and *u* under centralized decision-making with public and user supervision on total governance revenue is depicted in Fig 8. The curves show the trend of the effect of the public on the total revenue of governance and the trend of the effect of users on the total revenue of governance under centralized decision-making. At the moment of *t* = 10, when the increasing amount of parameter *s* and parameter *u* is the same, the user’s supervision increases the governance revenue by 4.13%, and the public’s supervision increases the governance revenue by 1.7%. In the case of centralized decision-making, user supervision has a more significant effect on the total revenue of governance.

As shown in Figs 7 and 8, the parameters *s* and *u* have a positive effect on the total revenue of governance. This indicates that the higher the level of supervision efforts by the public and users, the greater the total revenue, which shows that the level of supervision efforts by users has a greater impact on total revenue of governance than public supervision.

Users are the prominent participants in the exchange of resources on the platform. Because of the multilateral linkages between multiple stakeholders, user violations can be linked to the performance of a broader range of subjects with which they are associated. This linkage effect between stakeholders limits violations such as the unequal "exploitation" of platform corporate. Therefore, for users and the public in the governance of social responsibility of platform corporate, the governance utility is more reflected in the monitoring of each other by users and the monitoring of platform corporate.

**Fig 9:**
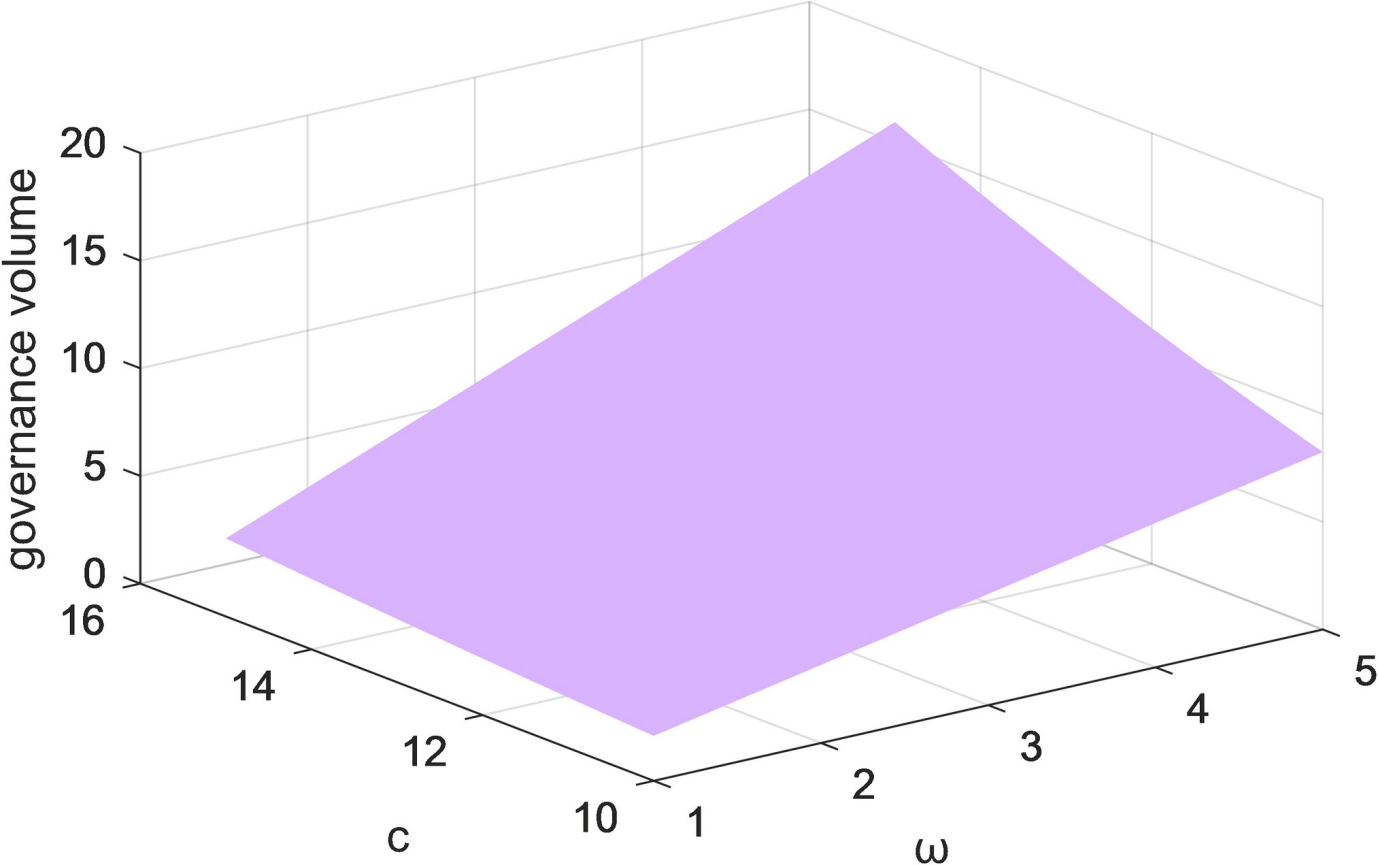
The effect of changes in parameters *c* and*ω*on the total governance volume under centralized decision-making without consideration of users and public scrutiny.

**Fig 10:**
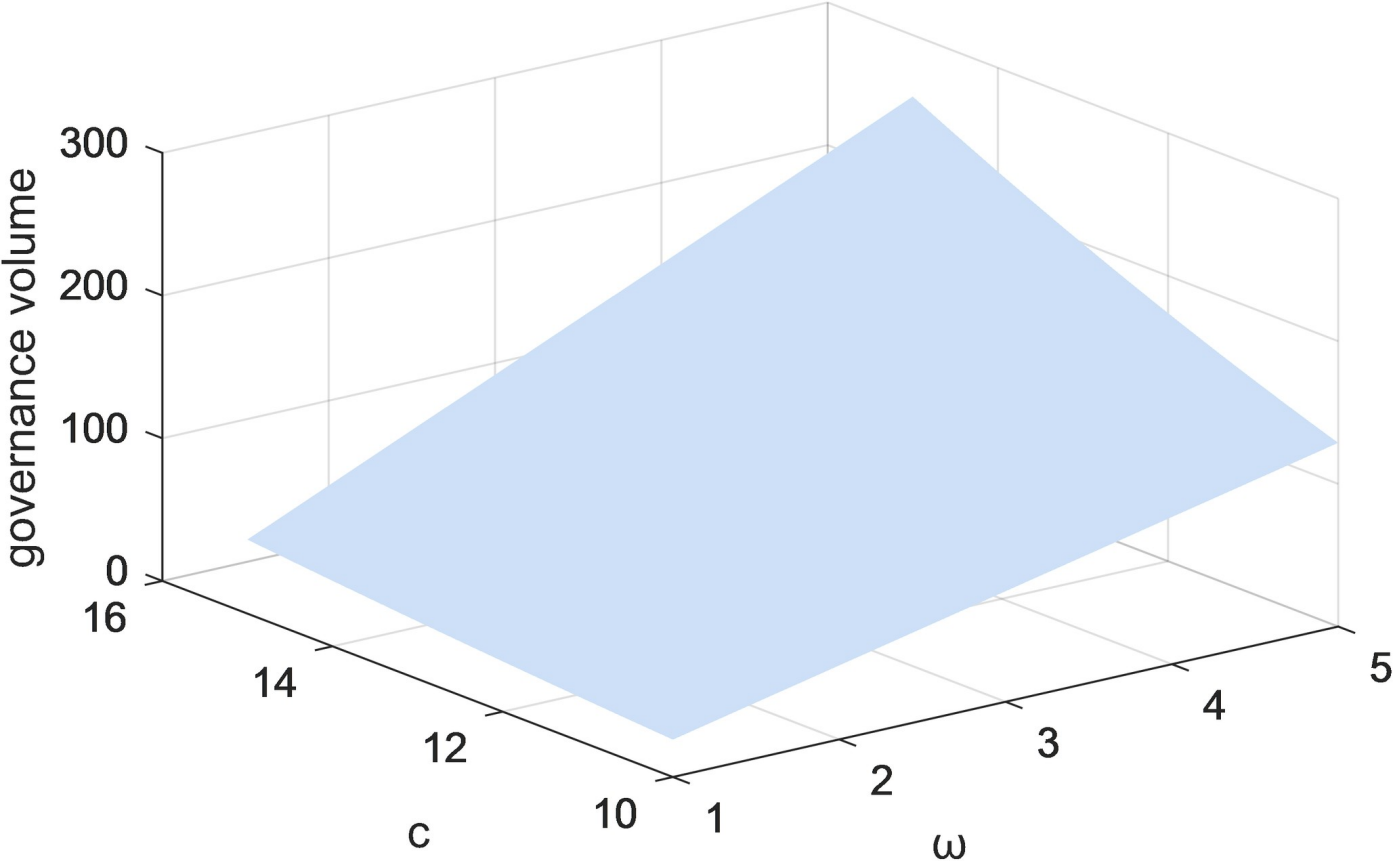
The effect of changes in parameters *c* and*ω*on the total governance volume under centralized decision-making with consideration of users and public scrutiny.

Figs 9 and 10 represent the variation of the two-parameter (c and ω) on the volume of governance in both cases, with and without considering users and public scrutiny under centralized decision-making. As the coefficient of influence of governance volume on governance benefits increases, the higher the governance volume. The results suggest that the volume of governance and the revenue of governance are mutually reinforcing from the perspective of the platform companies. To achieve governance of the lack of social responsibility and alienation, platform corporations need to build the essential functions of the platform and the rules for resource trading. The efforts made by platform enterprises in governance can create a beneficial governance atmosphere, thus improving the industry’s governance level.

**Fig 11:**
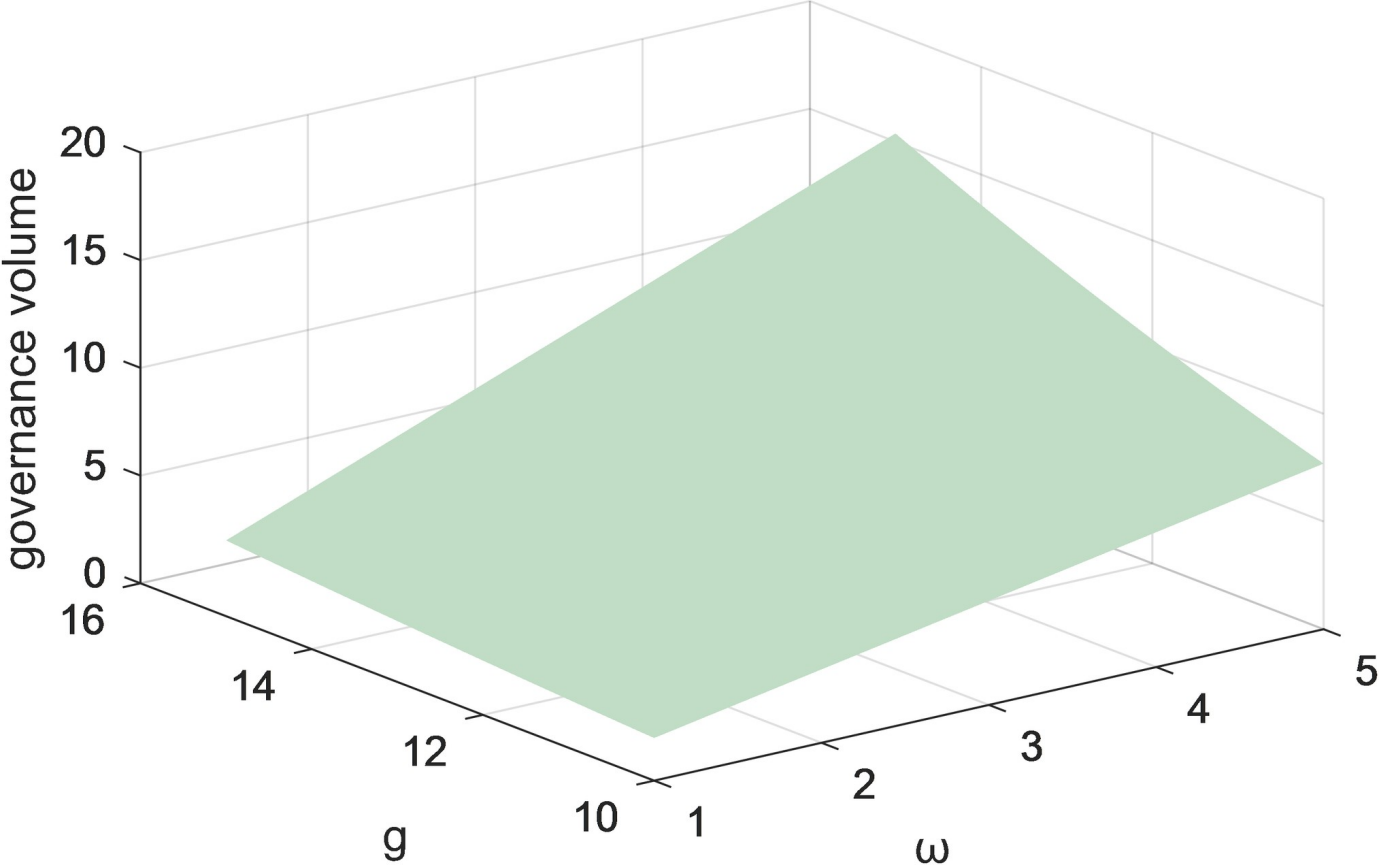
The effect of changes in parameters g and ωon the total governance volume under centralized decision-making without consideration of users and public scrutiny.

**Fig 12:**
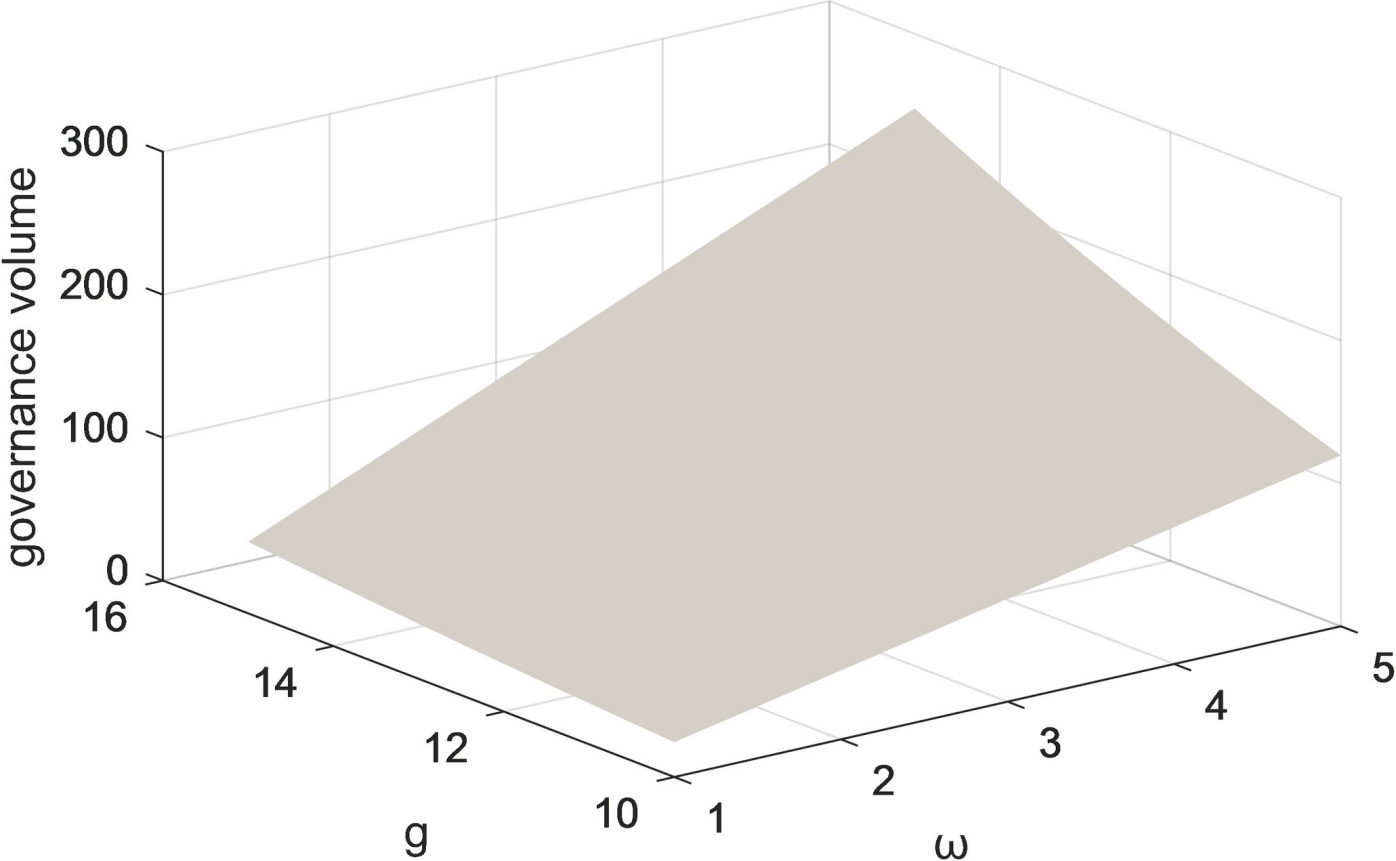
The effect of changes in parameters c and ωon the total governance volume under centralized decision-making without consideration of users and public scrutiny.

Figs 11 and 12 represent the variation of the two-parameter (g and ω) on the volume of governance under centralized decision-making with and without considering both user and public oversight. The results indicate that when considering the influence of two parameters, the growth of governance volume is higher. The government is the macro-controlling subject of social responsibility governance. The more the government invests financial resources and energy in formulating relevant laws, regulations, and policies, the more these measures can lay a perfect institutional foundation for platform corporate, thereby promoting the enhancement of governance volume. The improvement of governance volume will encourage the government’s enthusiasm for social responsibility governance, forming a virtuous circle. So the government needs to play a guiding role in incorporating multiple industries and types of subjects into the development of the governance system, with a common goal as the lead in realizing pluralistic co-governance.

Figs 9–12 show the impact of the coefficients of the effect of platform corporations and government departments and the amount of governance on governance benefits on the amount of governance when the centralized scenario is used as an example. When the coefficients of the impact of platform corporations and governments and governance volume on governance gains are higher, the governance volume is higher. Under centralized decision-making, the governance of socially irresponsible and alienating behaviors by platform firms and government departments can have an iterative effect on the effectiveness of governance. Taken together, this suggests users, the public, the corporation, and the government, all positively impact governance volume. Therefore, for the governance of platform CSR, it is necessary to give full play to the role of all participants and drive more stakeholders to participate.

## Conclusions

Platform economy has accelerated the flow and rational allocation of production factors, promoting upgrading the industrial chain and market globalization. With platform economy as the background, platform corporations are also developing rapidly in the 21st century, providing support in leading development and promoting innovation. Traditional corporations began to transform into platforms under the general digitalization trend, and their production efficiency was greatly improved. However, while platform corporations are meeting the needs of bilateral users, building platform ecosystems, and expanding their businesses, they also need to respond to social concerns and value the governance of social responsibility to achieve long-term development. Therefore, based on the analysis of platform corporate social responsibility’s content and governance subjects, this article constructs a mathematical model of platform corporate social responsibility governance using differential game theory. The following conclusions are drawn through theoretical analysis and model-solving:

Most of the current research on the social responsibility of platform corporations is based on Internet platform corporations, and other types of corporations [55, 56]. However, the characteristics of social responsibility vary among corporations in different industries. This study points out the characteristics of social responsibility of platform corporations, which refer to the organizations of traditional corporations that have undergone platform transformation. The social responsibility of platform corporations is characterized by two aspects. First character is that Platform CSR is more of a compliance responsibility. The platform corporation is to use data as an entry point and uses the optimization of data resources to achieve a win-win situation with partners. In addition, as the platform builder, the platform corporation realizes a reasonable exchange between platform users by formulating resource interaction rules. Therefore, the reasonable and legitimate utilization of data resources and the formulation of reasonable interaction rules are important aspect of ensuring the interests of collaborators. The second character is the diversity of governance subjects. Platform CSR needs to consider not only the companies’ self-consciousness under government constraints but also the need for external monitoring. Platform corporations focus on user needs and provide solutions for users, but driven by interests, platform corporations will steal user information when collecting user information, which damages users’ interests. In addition, the necessity of considering public supervision is because platform corporations will impact the public’s behavior habits and value orientation, and the public can supervise the possible negative impact on platform corporations.

Existing research has pointed out the relationship between corporate social responsibility and corporate performance [57], but has not discussed the dynamic changes in the governance of illegal and irregular behavior. Over time, the volume of governance and the benefits of socially responsible governance can eventually reach a specific stable value, and the benefits of governance decrease as the amount of governance increases. Therefore, social responsibility pursues social goals, and there is a conflict with business interests. If platform corporations want to achieve the goal of sustainable development, they must find the best combination of social responsibility and economic benefits. From the model results, the ultimate revenue of social responsibility governance is above zero, so governance behavior will increase benefits in the long run. In addition, the strengthening of social responsibility governance in platform corporations, the improvement of governance level, and the increase in the influence of corporations ultimately contribute to the rise in the benefits of social responsibility governance.

Some existing studies have pointed out that stakeholders (government and consumers, etc.) play an essential role in improving social responsibility governance performance [58], and the effectiveness of individual supervisors needs further discussion. In this study, it was pointed out that: Compared with the social responsibility governance that only considers the government and the platform corporation, the governance volume is significantly higher after considering public and user supervision than without supervision, which indicates that external supervision can lead to higher governance volume in social responsibility governance. When the level of monitoring effort of the public and users is the same, the level of monitoring effort of the users can cause a more significant increase in total benefits, indicating that the level of monitoring effort of the users has a more significant impact on the total benefits of governance compared with the monitoring of the public. It also shows that platform corporations are centered on the needs of their users and that the lack and alienation of social responsibility affect their users’ interests more directly. For the public, corporate social responsibility issues often involve the reputation of corporations, users, and the government. The impact generated is relatively small compared to the direct impact on users.

Another contribution of this article is to clarify the best way for platform corporate social responsibility governance. The centralized decision-making approach can result in greater governance volume and governance benefits, which also validates the idea of multiple centralized governances of social responsibility in the current study. The governance of platform corporate social responsibility needs to view the governance subject as a whole system and include stakeholders in the consideration of the study. By clarifying the status and functions of the governance subjects, it is possible to realize the multi-dimensional synergistic governance of the social responsibility of platform corporations.

## Theoretical implications

On the one hand, the social responsibility of traditional corporations under the transformation of platform transformation is taken as the starting point, and the content of platform corporation social responsibility is studied, which enriches the research on the connotation of different types of corporate social responsibility. The social responsibility of platform corporations includes compliance-based responsibility and duty-based responsibility. The social responsibility of platform corporations is mainly compliance-based responsibility, which means that platform corporations should create high-quality platforms, formulate access policies, and reasonably utilizing data to provide quality services for bilateral users. This definition enriches the research on the connotation of social responsibility of different types of corporations. On the other hand, it breaks through the internal [59, 60] and supply chain research perspectives of CSR to study the social responsibility governance of traditional corporations from the perspective of digital platform ecology. This perspective encompasses more governance subjects and explains the role of social responsibility governance subjects in the new economic situation. It responds to the scholars’ call for the plurality of governance subjects [61]. Socially responsible governance is the action of governments, platform companies, users, and the public based on the same overall goal. This collective and unified approach to action is a compelling validation of the theory of polycentric governance.

The differential game method is used to construct a game model for the governance of platform corporation social responsibility and explore the changing law of platform corporation social responsibility from the perspective of process continuity. In corporate social responsibility governance on platforms, the decisions of all parties involved in the game will affect the decisions of other stakeholders, and they will change in real time with the parameters of the governance entity. So, the research problem is transformed into the process of governance quantity and governance benefits changing from one state to another, ultimately obtaining the optimal decision result in a stable state. The influence of users and the public are considered in the model to explore the monitoring role of external agents, and the utility strength of users and the public is derived separately, which extends the study of the influence of the public [62] or users [63, 64] on social responsibility governance alone.

## Practical implications

The development of the digital economy has driven the transformation of traditional manufacturing corporations, which utilize digital platforms for resource exchange and innovation operational processes. However, with the increase of digital technology and platform openness, corporations have encountered social responsibility deficiencies and social responsibility alienation behaviors such as data abuse, which urgently require governance. This article first analyzes the content of social responsibility and governance subjects and then constructs a differential game model for the decision-making process based on the subject relationship. Obtain the optimal strategy through model-solving.

The decision-making approach of collaborative governance obtained in this article helps to solve the problem of choosing the social responsibility governance approach for platform corporations. It has been proven that the governance of social responsibility in platform corporations is fundamentally based on the collaboration of multiple rights centers and requires multiple governance entities to play their roles simultaneously. Therefore, it guides the government, platform corporations, users, and the public to cooperate in social responsibility governance. In addition, this study can provide a scientific basis and suggestions for the government and other governance entities, helping socially responsible governance entities play their respective roles better, thereby improving governance efficiency and quality. This study aims to provide a reference for the supervision of social responsibility and the formulation of relevant policies and promote the rational planning of social responsibility and the direction of commercial profit development.

## Management implications

To bring the governance effectiveness of the main subject into play and ensure the stable and healthy operation of the ecosystem of platform corporations, the following suggestions are made to the platform corporations, the government, users and public:

Government departments need to play the role of facilitator of the macro trust environment. A traditional company that has undergone a platform transformation, his social responsibility is more reflective of data governance. The governance boundary extended to the platform ecosystem, and the governance content is complex, so the governance content of platform CSR puts newer demands on government governance. In the face of the complex situation of social responsibility governance, the government needs to reconstruct the evaluation indexes of social responsibility and appropriately introduce third-party regulators. In addition, it should focus on critical aspects such as the entry and exit of platforms and the process of subject interaction and strengthen the penalties for serious violations. On the other hand, the government can set up a special fund to subsidize the participation of corporations and other subjects in fulfilling and supervising social responsibility to enhance the subjects’ enthusiasm for social responsibility governance.

The platform corporation is the leader of the platform ecosystem, and bilateral users on the supply and demand sides have close dependencies and interests with the platform corporation. When there is a value conflict in cooperation between platform users, opportunistic or speculative behaviors may arise. Therefore, platform corporations can establish a unified value goal to motivate users and establish an excellent order to fulfill their social responsibility. On the other hand, platform corporations can use their advantageous position to collaboratively integrate user resources and establish rules for resource interaction to avoid "value co-destruction" due to irrational competition for resources. For the autonomy of platform corporations, the main goal is to build a secure and efficient platform to ensure the legality of platform activities and prevent illegal and irregular behaviors such as information leakage.

Users and the public should play the role of supervisors. Users are the main subject of platform activities. Users, on the one hand, need to regulate their behavior. On the other hand, they need also fulfill the right to monitor. They can use various media to expose the lack of social responsibility and the alienation of social responsibility, which can supervise illegal and irregular behaviors in social responsibility governance. The general public can also share and learn about social responsibility knowledge actively assume feedback on social responsibility deficiencies and alienation behaviors, and form a sense of responsibility for society.

## Research limitations and future prospects

Although this study discusses the changing process of governance parameters and agent strategy selection from the perspective of multiple social agents, we believe that this study can be expanded from more aspects.

The platform transformation of traditional corporations has transformed their internal organizational structures to adapt to the new form of economy, resulting in a gradual blurring of the corporation’s boundaries. The platform transformation of the internal organizational structure of the corporation is mainly characterized by the decentralization of rights so that the corporation’s employees have greater autonomy. As a result, the corporation’s employees started to participate in the innovation activities as platform users, which mean the type of platform users is no longer limited to outside the corporation. In this study, the users were only treated as a unified, undifferentiated body. They were not categorized, while some studies have confirmed that employees’ backgrounds, emotions, and cognition [65, 66] closely relates to social responsibility. Therefore, future research could categorize users, consider corporate employees to be part of platform users, and study the influence of employees in the governance of social responsibility of platform corporations.

In the governance of platform corporate social responsibility, if the evaluation organizations make unreasonable evaluations to make private profits in cooperation with the subject of interest, it will also affect the social responsibility governance, In addition, some studies have pointed out that evaluation agencies have a regulating effect on corporate social responsibility governance [67, 68]. Therefore, in future research, evaluation agencies can be considered as one of the governance subjects to build a new model to study the role of more subjects in platform corporate social responsibility governance.
